# Mitochondria-enriched protrusions are associated with brain and intestinal stem cells in *Drosophila*

**DOI:** 10.1038/s42003-019-0671-4

**Published:** 2019-11-22

**Authors:** Sharyn A. Endow, Sara E. Miller, Phuong Thao Ly

**Affiliations:** 1 Programme in Neuroscience and Behavioural Disorders, Duke-NUS Medical School, Singapore, 169857 Singapore; 2 Department of Cell Biology, Duke University Medical Center, Durham, NC 27710 USA; 3 Department of Pathology, Duke University Medical Center, Durham, NC 27710 USA

**Keywords:** Quiescence, Microtubules, Neural stem cells

## Abstract

Brain stem cells stop dividing in late *Drosophila* embryos and begin dividing again in early larvae after feeding induces reactivation. Quiescent neural stem cells (qNSCs) display an unusual cytoplasmic protrusion that is no longer present in reactivated NSCs. The protrusions join the qNSCs to the neuropil, brain regions that are thought to maintain NSCs in an undifferentiated state, but the function of the protrusions is not known. Here we show that qNSC protrusions contain clustered mitochondria that are likely maintained in position by slow forward-and-backward microtubule growth. Larvae treated with a microtubule-stabilizing drug show bundled microtubules and enhanced mitochondrial clustering in NSCs, together with reduced qNSC reactivation. We further show that intestinal stem cells contain mitochondria-enriched protrusions. The qNSC and intestinal stem-cell protrusions differ from previously reported cytoplasmic extensions by forming stem-cell-to-niche mitochondrial bridges that could potentially both silence genes and sense signals from the stem cell niche.

## Abstract (web summary)

Endow et al. find that protrusions associated with quiescent neural stem cells and intestinal stem cells in Drosophila contain clustered mitochondria. Live imaging and drug treatment provide evidence that mitochondrial positioning is microtubule-dependent. The authors suggest the protrusions function in niche communication.

In adult mammalian brains, most neural stem cells (NSCs) remain in quiescence, a reversible nondividing and nondifferentiating state^1^. The balance between proliferation and quiescence is tightly regulated to ensure adequate neuronal regeneration without premature NSC depletion. Similarly, *Drosophila* NSCs, or neuroblasts (NBs), transit between proliferation and quiescence. Almost all *Drosophila* NSCs enter quiescence at the end of embryogenesis, forming qNSCs, and exit quiescence shortly after larval hatching^2–4^.

Several cellular factors have been identified in *Drosophila* that govern entry into or exit from quiescence by NSCs. Entry into quiescence is regulated by inhibitors of Hox gene expression^5^, the pseudokinase Tribbles^6^, and the transcription factor Prospero^7^. Exit from quiescence, also known as reactivation, requires the evolutionarily conserved InR/PI3K/Akt insulin signaling^4,8,9^ and Hippo kinase signaling pathways^10,11^. These cellular regulators, in turn, respond to external signals from the NSC niche. Resident neural glia secrete a number of factors that control NSC reactivation, e.g., insulin-like-peptides^4,8,9^, and synchronize NSC reactivation through gap junctions and calcium oscillations^12,13^. However, other extrinsic cues that regulate NSC reactivation remain unexplored. Reactivation of qNSCs is essential for normal brain development—defects delay neurodevelopment and result in reduced brain size^10,14^.

Larval qNSCs display a characteristic cellular protrusion, which was first described more than 30 years ago^2^, although its cytological structure and function have been elusive. The protrusion forms when an embryonic NSC enters quiescence and retracts upon stem cell reactivation. qNSC protrusions have been reported to form junctions with the neuropil, interstitial brain regions containing axons, dendrites, and glial cell processes with relatively few cell bodies^2^. The neuropil could contribute to qNSC cellular function, potentially comprising a niche component. Stem cells and their niches^15,16^ have generated considerable interest because of their importance in tissue formation and self-renewal.

Cytoplasmic extensions or protrusions, including cytonemes^17^, tunneling nanotubes (TnTs)^18^, and the better known cilia and flagella^19^, are found on most or all cells. These cellular structures represent unconventional cytoplasmic compartments associated with specific functions, such as transport of signaling molecules between cells, movement of organelles, or other cytoplasmic components from one cell to another, and sensing of extracellular signals^11^. Specialized cellular extensions have also been identified, such as embryonic filopodia, which are required for cell elongation^20^. The finding of microtubule-based nanotubes on *Drosophila* male germline stem cells^21^—a new class of protrusions thought to mediate niche-stem-cell signaling interactions—has established their importance in stem cell maintenance and function.

Here we present new findings regarding the structure and possible function of larval qNSC protrusions based on ultrastructural analysis, fluorescence microscopy, and live imaging. We show that the qNSC protrusions are enriched in mitochondria and contain microtubules that exhibit forward-and-backward growth that could cluster the mitochondria and maintain their distribution. We further show that other insulin-sensitive stem cells—*Drosophila* midgut intestinal stem cells (ISCs)—contain mitochondrial-rich protrusions. The structural features of the stem cell protrusions that we report here have functional implications that may be important in stem cell quiescence and activation.

## Results

### Ultrastructural analysis of qNSCs

**Fig. 1:**
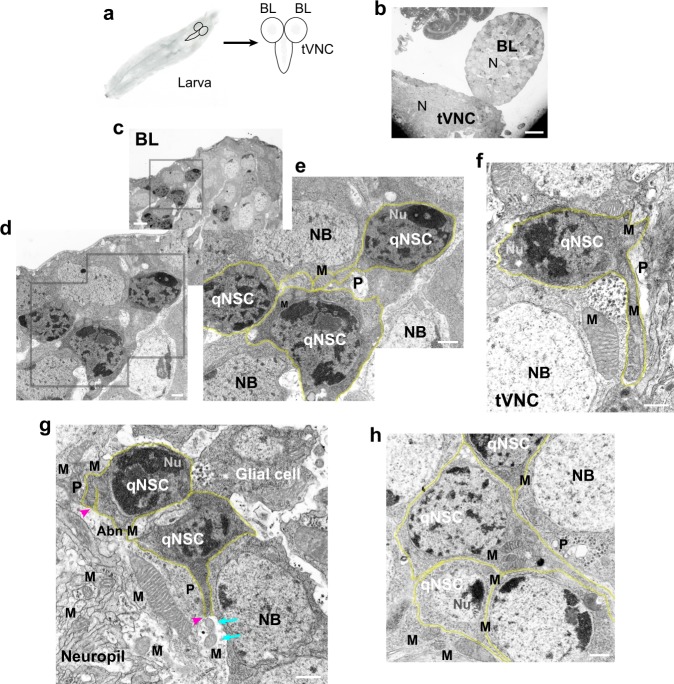
*Drosophila* larval brain qNSC protrusions contain clustered mitochondria as observed by TEM. **a** *Drosophila* larval brain. Larva, left; brain, right (schematic diagrams). Neuropil, gray regions in the brain lobe (BL) and thoracic ventral nerve cord (tVNC). **b**–**h** TEM images, amino-acid-depleted conditions. **b** Larval brain, low magnification. N, neuropil. **c** BL region (inset, gray box) at higher magnification in **d** and **e**, showing qNSCs (**e**, yellow overlays). **f** tVNC with mitochondria (M) in protrusions (P). **g** qNSC protrusions form junctions with the neuropil (magenta arrowheads); small mitochondria (cyan arrows) in the neuropil near a qNSC protrusion end. Putative glial cell (upper right) with an irregular cell body. Abn M, abnormal mitochondria (see Supplementary Fig. 1). **h** Clustered mitochondria in a qNSC protrusion neck (center). NB, neuroblast; Nu, nucleolus. Bars: 10 µm (**b**); 2 µm (**c**); 500 nm (**d**–**h**).

Because of the unknown nature of the qNSC protrusions, we examined their ultrastructure by transmission electron microscopy (TEM; Fig. 1). First instar larval brains (LBs), which consist of two brain lobes (BLs) and a thoracic ventral nerve cord (tVNC) (Fig. 1a), were enriched for qNSCs by hatching embryos on amino-acid-depleted food, then they were fixed, stained with tannic acid and OsO_4_, embedded and thin sectioned, and stained with uranyl acetate/lead citrate (Fig. 1b). TEM images showed cells with darkly staining nuclei containing large heterochromatic patches and a prominent nucleolus^22^, typical of larval qNSCs^23^ (Fig. 1c–h). The cells had little cytoplasm, irregular cell margins, and a cytoplasmic protrusion that was continuous with the cell membrane. These cells were identified as qNSCs based on the generally accepted correlation between silent or quiescent genes and heterochromatin^22^, and the presence of a single cellular protrusion, which is found on qNSCs, but not on other neural cells, such as glia^6^.

The qNSCs showed other large differences in cytology and morphology compared with glial cells, which were identified by their small, palely staining nuclei and the absence of large, darkly staining nuclear patches as observed by TEM^24^. Glia contain more cytoplasm and have a stellate appearance with several radiating processes^8,24^, rather than a single protrusion such as qNSCs^2^ (Fig. 1g). The cells with much larger, more uniformly staining nuclei were identified as NBs^2,24^ (Fig. 1e–h).

The qNSC protrusions varied greatly in diameter along their length, ranging from ~ 80–1300 nm, although their average diameter (323 ± 161 nm, mean ± SD, *n* = 27) was not significantly different for BL and tVNC qNSC protrusions (*P* = 0.2154, unpaired *t*-test).

Closer examination of qNSCs at high magnification revealed mitochondria clustered around the nucleus or in the neck—the region of the cell body where the protrusion begins—or packed along the protrusions, causing bulges and unevenness in their diameter (Fig. 1e–h) (*n* = 32, total = 34; *n* = 9 BLs + 5 tVNCs). qNSC protrusions that could be followed in the same plane of focus along their length (*n* = 9, total = 34) formed junctions with a region identified as the neuropil^2^, which contained numerous mitochondria (Fig. 1g). Most of these qNSCs displayed mitochondria along the protrusion length and near the protrusion junction with the neuropil (*n* = 8, total = 9), as though the mitochondria were being shed into the neuropil. A few qNSC protrusions that formed junctions with the neuropil (*n* = 2, total = 9) were adjacent to small mitochondria in the neuropil, as though the mitochondria had just been released from the protrusion into the neuropil (Fig. 1g, cyan arrows). The TEM images also showed atypical or distorted mitochondria with vacuoles or abnormal cristae^25^ in qNSC protrusions or cell bodies (*n* = 5, total = 34) (Fig. 1g and Supplementary Fig. 1). Apoptotic mitochondria are associated with mitochondrial biogenesis and cell death^26^.

Upon reactivation, qNSCs enlarge to form NBs, which contained mitochondria that were sometimes positioned close to the nucleus (*n* = 7, total = 44; *n* = 6 BLs + 4 tVNCs) (e.g., Fig. 1f, right), but mitochondria were not clustered around the NB nuclei, as observed for qNSC nuclei. The absence of clustered mitochondria around NB nuclei implies that the mitochondria undergo redistribution after qNSC activation (*n* = 37, total = 44). As mitochondrial motility in other cells is dependent on microtubules and kinesin motors^27^, we looked for microtubules in the qNSCs that could underlie the dispersion of mitochondria after activation. However, the qNSCs did not contain long filaments by TEM that could be identified with certainty as microtubules.

### Fluorescence microscopy of NSC protrusions and cell bodies

**Fig. 2:**
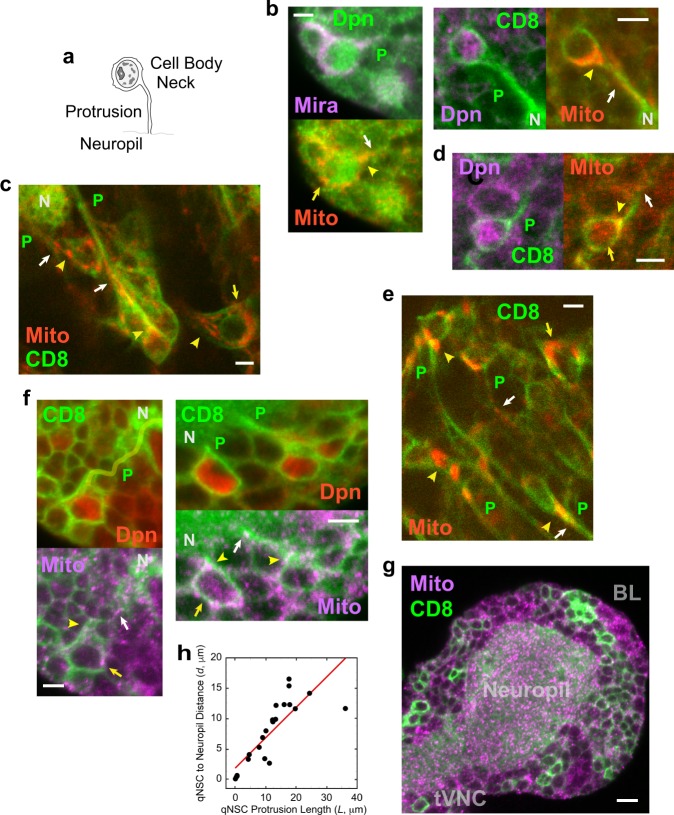
*Drosophila* larval brain qNSC protrusions contain clustered mitochondria as observed by fluorescence microscopy. **a** qNSC (schematic drawing). Nucleus, gray; nucleolus and heterochromatin, dark patches. **b** Mito-RFP (Mito, red) in BL qNSC protrusions, necks, and cell bodies (left, fed larva; right, starved larva). P, protrusion; CD8, CD8 membrane protein; Dpn, Deadpan; Mira, Miranda; N, neuropil. Mitochondria in qNSC protrusions (white arrows), qNSC necks (yellow arrowheads) and qNSC cell bodies (yellow arrows). **c** Mito-RFP in live BL NSCs (fed larva); **d** Mito-RFP in tVNC qNSC (fed larva). **e** Mito-RFP in live tVNC NSCs (fed larva). **f** BL qNSCs immunostained for TOMM20 mitochondrial protein (Mito, purple; left, fed larva; right, starved larva). Protrusion, left top, yellow overlay. **g** Neuropil immunostained for mitochondria by TOMM20 antibody. Bars: 3 µm (**b**–**f**); 6 µm (**g**). **h** qNSC distance to neuropil (*d*) vs. protrusion length (*L*); data points fit to *d* = 1.8078 + 0.49838* L* (*n* = 24; *R*, correlation coefficient = 0.823).

The presence of clustered mitochondria in qNSC protrusions was confirmed by immunostaining larval brains expressing a mitochondrial protein fused to red fluorescent protein (RFP), referred to here as mito-RFP, induced by a NSC-specific driver, *insc-gal4* (Fig. 2). qNSCs in larvae hatched on normal or amino-acid-depleted food, denoted “fed” or “starved,” respectively, were identified by staining by a NSC-specific transcriptional factor, Deadpan (Dpn) and the presence of a cellular protrusion (Fig. 2a), which was visualized by *insc-gal4*-induced CD8-GFP, a membrane protein, or, in some experiments, by immunostaining with the neural progenitor-specific scaffold protein, Miranda (Mira). The qNSCs identified by fluorescence microscopy showed features that overlapped with those identified by TEM.

Dpn-positive qNSCs containing a protrusion showed mito-RFP-labeled mitochondria distributed along the thin cytoplasmic protrusion and clustered in the neck and cell body (fed, *n* = 25 Dpn^+^ protrusion^+^ mito-RFP^+^ qNSCs, *n* = 3 BLs; starved, *n* = 19 Dpn^+^ protrusion^+^ mito-RFP^+^ qNSCs, *n* = 7 BLs; Fig. 2b). There was no significant difference between fed and starved larvae in *insc-gal4*-induced mito-RFP mean fluorescence intensity in BL *z*-sections recorded on the same day or next day using identical settings. In addition, mito-RFP distribution did not differ significantly between fed and starved larval BL *z*-sections, as indicated by minimum and maximum fluorescence values (Supplementary Fig. 2). Live imaging of NSCs expressing mito-RFP and CD8-GFP, both induced by *insc-gal4*, also showed mitochondria in NSC protrusions and necks, and packed in cell bodies (Fig. 2c and Supplementary Fig. 2). Further, qNSCs in tVNCs, as well as BLs, showed mitochondria distributed along protrusions and clustered in cell bodies by mito-RFP fluorescence in fixed or live larval brains (Fig. 2d, e and Supplementary Fig. 2).

Mito-RFP localization in qNSCs was confirmed by staining larval brains with antibodies (Abs) specific for a mitochondrial outer membrane protein, TOMM20 (fed, *n* = 21 Dpn^+^ protrusion^+^ TOMM20^+^ qNSCs, *n* = 4 BLs + 2 tVNCs; starved, *n* = 20 Dpn^+^ protrusion^+^ TOMM20^+^ qNSCs, *n* = 6 BLs + 2 tVNCs) (Fig. 2f and Supplementary Fig. 2). The TOMM20 Ab-stained larval brains also confirmed that the neuropil is rich in mitochondria (Fig. 2g), as indicated by TEM.

The qNSC protrusions entered the neuropil or formed junctions with the neuropil (Fig. 2 and Supplementary Fig. 2), as reported previously^2^. The qNSCs that were farther from the neuropil had longer protrusions and those that were closer had shorter protrusions. The shortest protrusions were <1 µm in length (*n* = 5, total = 24) from the cell body to the neuropil (e.g., Fig. 2f, right, qNSC at left), whereas the longest protrusions were ~16–36 µm in length (*n* = 7, total = 24) (e.g., Fig. 2c and Supplementary Fig. 2b, qNSC at left). There is a strong positive correlation between qNSC protrusion length, *L*, and qNSC distance to the neuropil, *d*, with *d* = 1.8078 + 0.49838*L*, *R* = 0.82312 (Fig. 2h). The protrusion length dependence on distance to the neuropil implies that the neuropil is a niche component that may be involved in maintaining qNSCs, possibly by serving as a reservoir for mitochondrial exchange.

The qNSC protrusions differ from previously described cytoplasmic processes, such as cytonemes and TnTs, in that they contain mitochondria along their length. They share similarities with TnTs, reported previously as containing mitochondria^18^, in that their diameter (~80–1300 nm) also overlaps with that of TnTs (~50–200 nm). However, a major difference between the qNSC protrusions and TnTs is that the qNSC protrusions are not cell-to-cell bridges such as TnTs. Instead, the qNSC protrusions are present on stem cells and form connections to a putative niche component, the neuropil—in this regard, they share features with male germline stem cell nanotubes^21^. However, the qNSC protrusions are strikingly different from nanotubes in that they form mitochondrial bridges that connect the stem cells to their putative niche. Further, NSCs have only a single protrusion, whereas the majority of male germline stem cells display multiple nanotubes^21^.

### Microtubule dynamics in NSC protrusions

**Fig. 3:**
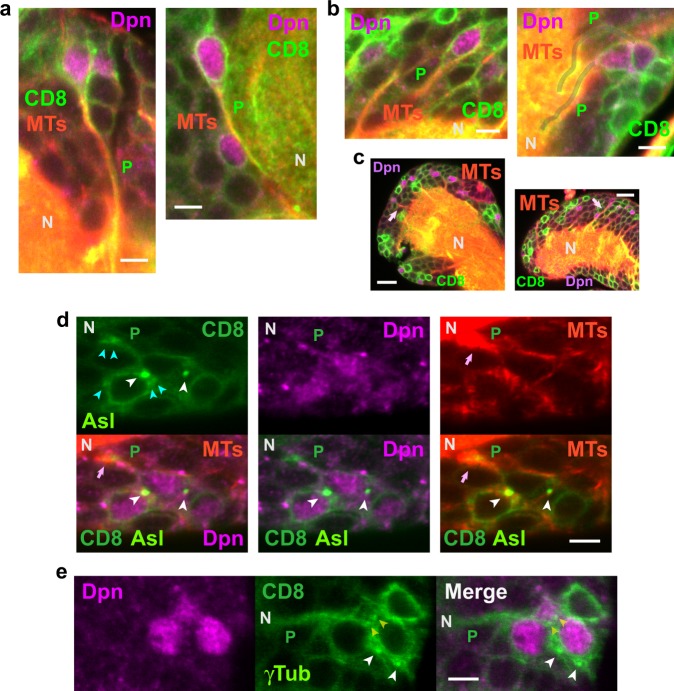
*Drosophila* larval brain qNSC protrusions contain microtubules and centrosomes as observed by immunofluorescence microscopy. **a** BL qNSC protrusions (left, fed larva; right, starved larva) and **b** tVNC qNSC protrusions (left, fed larva; right, starved larva) are stained by αTub antibody, indicating the presence of microtubules (MTs) in the protrusions. Protrusion (P), right, gray overlays. **c** Spikes of αTub fluorescence (pink arrows), corresponding to microtubules, from the neuropil (N) into BL (left) and tVNC (right) qNSC protrusions (fed larvae). **d**, **e** Centrosomes (white arrowheads) associated with qNSC cell body nuclei (fed larvae). Centrosomes in other focal planes (**d**, cyan arrowheads) or the same focal plane (**e**, yellow arrowheads) associated with other NSC cell bodies. Microtubule spike (pink arrows) in qNSC paired or bundled protrusions. Asl, YFP-Asl centriole protein; γTub, γ-tubulin-GFP. Bars: 3 µm (**a**, **b**, **d**, **e**); 10 µm (**c**).

We also stained larval brains for tubulin to determine whether microtubules were present in qNSC protrusions that could underlie the inferred dispersion of mitochondria after qNSC activation (Fig. 3). αTub immunostaining showed fluorescence along qNSC protrusions (Fig. 3a, b) and in the neuropil with spikes of αTub fluorescence emanating from the neuropil into protrusions (Fig. 3c), as though microtubules were growing from the neuropil into the protrusions. Staining of larval brains with the YFP-Asl centrosomal protein^28^ showed small bright fluorescent spheres associated with the nuclei of qNSCs (*n* = 35 Dpn^+^ protrusion^+^ Asl^+^ qNSCs, *n* = 3 BLs) that were identified as centrosomes based on their size and appearance, and the specificity of the YFP-Asl protein^28^ (Fig. 3d). This was confirmed by fluorescence labeling of qNSCs with γ-tubulin Abs^29^ or γTub-GFP^30^ (*n* = 27 Dpn^+^ protrusion^+^ γTub^+^ qNSCs, *n* = 10 BLs) (Fig. 3e). YFP-Asl-positive qNSC protrusions were also labeled by α-tubulin Ab along the length of the protrusion (*n* = 31 Dpn^+^ protrusion^+^ Asl^+^ αTub^+^ qNSCs, total = 35) or within the protrusion near its junction with the neuropil (*n* = 3 Dpn^+^ protrusion^+^ Asl^+^ αTub^+^ qNSCs, total = 35), indicating the presence of microtubules in the protrusions (Fig. 3d).

**Fig. 4:**
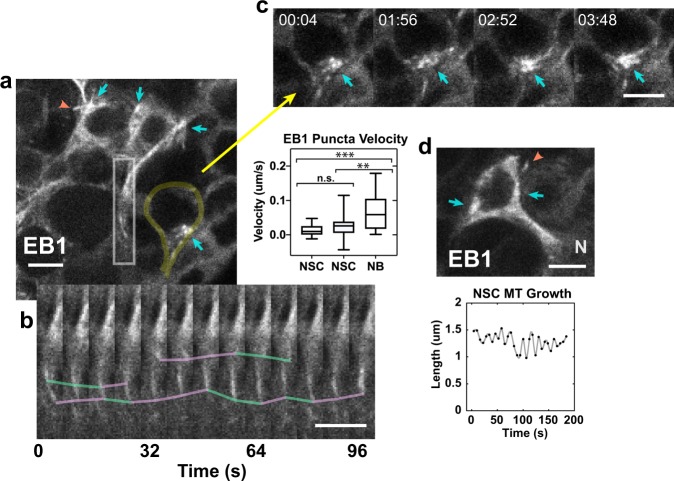
Microtubules in NSC protrusions are nucleated by centrosomes and show back-and-forth movement as analyzed by EB1 tracking. **a** Live imaging of EB1-EGFP shows microtubule nucleation by centrosomes (cyan arrows) in CD8-GFP-labeled NSCs. Microtubule growing outside the cell body (orange arrowhead; Supplementary Movie 1). NSC at the lower right, yellow overlay. Inset (bottom), **b** kymograph showing slow movement of EB1-EGFP puncta down protrusions, indicating microtubule growth (cyan lines), alternating with pauses and slow backward movement (purple lines). Plot, middle, EB1 puncta velocity in NSCs (0.013 ± 0.004 µm/s, mean ± SEM, *n* = 3 NSCs, 20 tracks, 123 steps; 0.025 ± 0.006 µm/s, *n* = 3 NSCs, 23 tracks, 69 steps) and NBs (0.059 ± 0.009 µm/s; *n* = 3 NBs, 30 tracks, 45 steps). The two NSC EB1 puncta velocity peaks were not significantly different when track velocities were compared (*P* = 0.1146, two-tail *t*-test), but the NSC velocities differed significantly from NB puncta mean velocity (*P* = 0.0002 and *P* = 0.0049, respectively, for the slower and faster NSC track velocities, two-tail *t*-tests); the NB velocities overlapped EB1 mean velocity reported previously for S2 cells (0.063 ± 0.015 µm/s)^31^. **c** Fragmentation and coalescence of centrosome (cyan arrows) over time. Time, min:s. **d** Microtubule growing from within a NSC to outside the cell body (top, orange arrowhead; centrosomes, cyan arrows). N, neuropil. Plot (bottom), NSC microtubule length fluctuations over time, paralleling microtubule growth in NSC protrusions (**a**, inset). Bars, 3 µm.

We imaged live NSCs expressing CD8-GFP to delineate NSC bodies and protrusions, together with the microtubule plus-end binding protein, EB1-EGFP, to visualize microtubule growth, both driven by *insc-gal4* (Fig. 4). The live imaging revealed that microtubule nucleation in NSCs was from centrosomes that were associated with the nucleus in the cell body; microtubules grew across and around the nucleus, with slow growth down NSC protrusions towards the neuropil, alternating with slow backward movement towards the cell body (Fig. 4a, b and Supplementary Movie 1). The alternating forward-and-backward movement resulted in an unusual back-and-forth flow, differing from the rapid EB1 movement away from the centrosome that we observed in activated NBs during asymmetric division (Supplementary Movie 2) and attributed to rapid microtubule growth^31^.

The net forward velocities of EB1 puncta in NSC protrusions formed two peaks of 0.013 ± 0.004 µm/s and 0.025 ± 0.006 µm/s, which may represent NSCs in different stages of quiescence or activation. NSCs with EB1 puncta moving at these two different velocities, corresponding to different microtubule growth rates, were observed adjacent to one another in the same larval brains. These EB1 puncta velocities were ~ 2–4-fold slower than those in activated, asymmetrically dividing NB spindles (0.059 ± 0.009 µm/s) (Fig. 4a, plot) in the same field (Supplementary Movie 2), which overlap with those reported previously for *Drosophila* S2 cells (0.063 ± 0.015 µm/s)^31^.

The unusually slow forward-and-backward EB1 puncta movement in NSC cell bodies and protrusions that we observed, corresponding to microtubule growth away from and towards centrosomes in the cell bodies, respectively, differs from the rapid elongation and shrinking by plus ends that characterizes microtubule growth in other cells, referred to as dynamic instability^32^. Microtubule growth towards the cell body could account for the microtubule spikes that project into the NSC protrusions from the neuropil (Fig. 3).

Microtubule growth in NSCs was also unusual in that centrosomes in the cell bodies appeared to undergo fragmentation and coalescence (Fig. 4c and Supplementary Movie 1)—this was also observed in activated NBs during division (Supplementary Movie 2). Further, microtubules were sometimes observed that were nucleated by centrosomes in the NSC body but appeared to grow outside the cell (Fig. 4a, d and Supplementary Movie 1). The slow back-and-forth growth of these single microtubules paralleled the slow microtubule growth in NSC processes inferred from EB1 tracking—it is also similar to the back-and-forth movement or flux by stationary mitochondria that do not move directionally along axonal microtubules in vivo^33^. Thus, mitochondria in qNSC cell bodies and protrusions may be maintained in position by back-and-forth microtubule growth. Activation of qNSCs could induce rapid microtubule growth, as observed in activated NBs, causing mitochondria to move and redistribute in NSC bodies and protrusions.

### Mitochondria clustering in Taxol-treated larval brains

The effect of suppressing microtubule dynamics on mitochondria in NSCs was tested by treating embryos and larvae with the microtubule-stabilizing drug Taxol. Larvae that were hatched and maintained on medium containing 100 µM Taxol developed more slowly than normal, reaching the late L1/L2 larval stage 2 days after hatching, rather than 1 day after hatching (*n* = 5 tests; control, *n* = 3 tests). Taxol treatment (~ 80–100 embryos/test) also reduced survival to the mature L3 larval stage and subsequent development (4.8 ± 4.3 mature L3 larvae, pupae, or adults per test, mean ± SD, *n* = 5 tests) compared with controls (*n* = 29 ± 9.8 mature L3 larvae, pupae, or adults per test, *n* = 3 tests). This significantly reduced frequency (*P* = 0.0026, unpaired *t*-test) shows that the Taxol treatment we used reduces embryo and larval viability, as well as delays larval development.

**Fig. 5:**
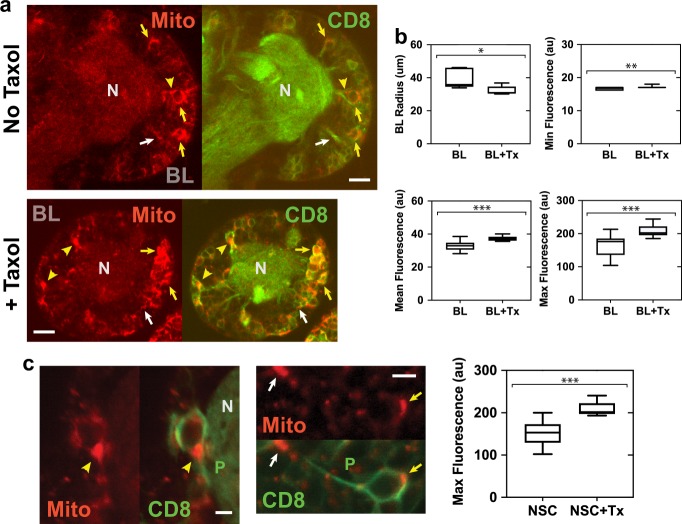
Mitochondria in Taxol-treated larval brains show enhanced clustering as observed by fluorescence microscopy. **a** Live imaging of mitochondria (Mito, mito-RFP, red) in control (top) and Taxol-treated (bottom) CD8-GFP-labeled (CD8, green) BL *z*-sections. Images were recorded using identical acquisition settings on the same day or next day. Mitochondria in qNSC protrusions (white arrows), necks (yellow arrowheads), or cell bodies (yellow arrows). BL, brain lobe; N, neuropil. Bars, 10 µm. **b** Taxol treatment (BL + Tx) resulted in significantly reduced brain radius (*r*_T_ = 32.2 ± 2.7 µm, mean ± SD, *n* = 5 BLs) compared with controls (BL; *r*_C_ = 39.0 ± 5.5 µm, *n* = 7 BLs; top left; *, *P* = 0.0308, unpaired *t*-test). Minimum fluorescence (Min, top right) differed by a small, but significant amount between Taxol-treated BLs (17.1 ± 0.1 au, mean ± SEM, *n* = 15 *z*-sections) and control BLs (16.7 ± 0.1 au, *n* = 20 *z*-sections; **, P = 0.0052, unpaired *t*-test); mean fluorescence (bottom left; Taxol-treated BLs, 37.4 ± 0.4 au, *n* = 15 *z*-sections; control BLs, 33.1 ± 0.6 au, *n* = 20 *z*-sections) and maximum fluorescence (Max, bottom right) were highly significantly increased after Taxol treatment (208.7 ± 4.7 au, *n* = 15 *z*-sections; controls, 166.5 ± 6.8 au, *n* = 20 *z*-sections; ***, *P* < 0.0001, unpaired *t*-test). au, arbitrary units. **c** Mito-RFP fluorescence in the NSC neck (left, yellow arrowhead) and protrusion (right, white arrow) after Taxol treatment. Bars, 3 µm. Plot (right), mito-RFP fluorescence in NSCs after Taxol treatment (NSC + Tx; 207.7 ± 3.2 au, mean ± SEM, *n* = 18) was highly significantly increased, compared with controls without Taxol (NSC; 151.5 ± 6.1 au, *n* = 22; ***, *P* < 0.0001, unpaired *t*-test).

The brains of 2-day Taxol-treated larvae were noticeably smaller than those of controls. Their smaller size was confirmed by live-imaging Taxol-treated late L1/L2 larval brains on the second day after hatching by recording *z*-stacks of CD8-GFP and mito-RFP fluorescence, and comparing the images with those from controls at the same stage acquired on the first day after hatching. The area of the section of each stack in which the BL was the largest was measured and the relative size of Taxol-treated and control BLs was estimated by modeling the BLs as spheres and the *z*-sections as circles, and calculating the average BL radius and volume for each group from the measured area. Two-day Taxol-treated larvae showed an average BL radius that was ~80% that of control larvae (Fig. 5a, b). These values correspond to a relative volume of ~55% for Taxol-treated larval BLs compared with controls. The smaller average radius of Taxol-treated larval BLs is statistically significant. These data show that Taxol affects development of the larval brain, causing it to be significantly reduced in size relative to controls.

Mito-RFP fluorescence in *z*-stack images was also measured and showed a small but significant increase in minimal fluorescence between Taxol-treated BLs and controls (Fig. 5b). The total mean fluorescence of Taxol-treated BLs was increased by a small amount that was highly significant compared to controls. Strikingly, the maximum fluorescence was greatly increased in Taxol-treated BLs compared with controls and this increase was highly statistically significant. The relatively small increase in total mean fluorescence of Taxol-treated BLs, together with the much larger increase in maximum fluorescence, indicate that the increased maximum fluorescence in the Taxol-treated BLs is due to a difference in distribution of the mitochondria, rather than a substantial increase in mitochondria after Taxol treatment.

We tested this idea by analyzing the regions of brightest mito-RFP fluorescence in images from Taxol-treated and control BLs. The brightest mito-RFP regions were found to localize to NSC cell bodies, necks, and protrusions, which could be identified by CD8-GFP membrane protein labeling (Fig. 5a, c). Quantification of the brightest mito-RFP-labeled NSC regions in Taxol-treated and control BL sections showed highly significantly increased mito-RFP fluorescence in NSCs of Taxol-treated BLs, compared with controls, with mean values close to those for the increased maximum fluorescence in Taxol-treated BLs and controls. These results show that there is a small increase in total mean mitochondrial fluorescence after Taxol treatment, but there is a large increase in mito-RFP maximum fluorescence in Taxol-treated BLs that is attributable to increased mitochondria in NSC cell bodies, necks, and protrusions, caused by a change in distribution of the mitochondria. Mitochondria are still clustered in NSC cell bodies and necks (Fig. 2b–f), and present in protrusions after Taxol treatment (Fig. 5a, c), but mitochondrial clusters in Taxol-treated NSCs are highly significantly brighter (Fig. 5c). These results indicate that stabilizing microtubules using Taxol causes a change in distribution of mitochondria in BLs, presumably by redistributing mitochondria from other regions of the larval brains, resulting in enhanced mitochondrial clustering in NSCs.

**Fig. 6:**
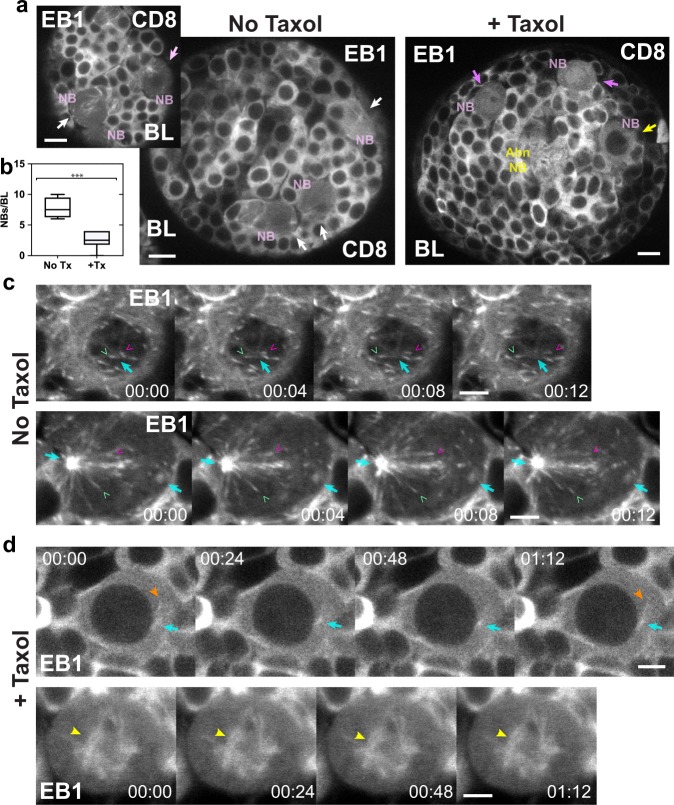
Taxol suppresses microtubule growth and elongation in larval brain NBs. **a** EB1-EGFP and CD8-GFP-labeled control (No Taxol) and Taxol-treated (+Taxol) BLs. Activated NBs (NB, pink) in control BLs (left) in prophase (top left, pink arrow) or mitosis (white arrows). NBs in Taxol-treated BLs (right) in prophase (yellow arrow) or mitosis (magenta arrows). Abnormal NB (Abn NB, yellow). **b** Plot, NBs in Taxol-treated BLs (+Tx; 2.5 ± 1.35 NBs/BL, mean ± SD, *n* = 25, *n* = 10 BLs) are reduced in frequency compared with controls (No Tx; 7.75 ± 1.71 NBs/BL, *n* = 31, *n* = 4 BLs; ***, *P* < 0.001, unpaired *t*-test). **c** Time-lapse images of control NBs in prophase (top) with the centrosome (cyan arrows) slightly above or below the plane of focus, or mitosis (bottom), showing rapid growth of single microtubules (green and magenta v’s; Supplementary Movies 3 and 4). **d** Taxol-treated NBs in prophase (top) with a small centrosome and faintly labeled microtubules (orange arrowheads) near the nucleus (black sphere) or in mitosis (bottom) with a rudimentary spindle (yellow arrowheads) and no growing or elongating microtubules (Supplementary Movies 5 and 6). Time, min:s. Bars, 6 µm (**a**); 3 µm (**b**, **c**).

We confirmed that Taxol suppresses microtubule dynamics under the conditions that we used by imaging 2-day Taxol-treated L2 larval brains expressing EB1-EGFP and CD8-GFP. EB1-EGFP puncta were difficult to visualize and image in the Taxol-treated larval brains, presumably because Taxol stabilizes microtubules, suppressing the formation of EB1 puncta, which form on growing microtubule plus ends^31^, and it also bundles microtubules^34^, which would make the puncta difficult to visualize. Faint puncta that could be discerned in some time-lapse images showed diffusion-like movement, but we did not observe forward-and-backward directional displacements such as those in NSCs without Taxol treatment. However, there was a striking effect of Taxol on microtubule growth and elongation in NBs, which form upon qNSC reactivation and divide asymmetrically^35^. Large, activated NBs were present in larval brains after 2-day Taxol treatment (Fig. 6a), but at a lower frequency than in 1-day control larval brains (Fig. 6b). This ~3-fold difference in frequency is highly statistically significant and indicates a reduced rate of qNSC reactivation upon Taxol treatment. This is likely important developmentally, given that defective NSC activation leading to reduced neurogenesis has been implicated in neurodegenerative diseases in adult mammalian brains^36^.

We further found that microtubule dynamics are suppressed in NBs after Taxol treatment (Fig. 6c, d and Supplementary Movies 3–6). Microtubule growth and elongation were not observed in NBs from Taxol-treated larval brains (*n* = 0, total = 19), but were observed in NBs from controls (*n* = 18, total = 18). This difference between Taxol-treated and control larval brains is highly statistically significant (*P* < 0.0001, *χ*^2^-test, Fisher’s exact two-tailed *P*-value). NBs in larval brains after Taxol treatment were arrested in prophase (*n* = 8, total = 19) or mitosis (*n* = 11, total = 19) with microtubule arrays that were uniformly labeled with EB1-EGFP (Fig. 6d), rather than with EB1 puncta characteristic of growing microtubule ends (Fig. 6c). The prophase- and metaphase-arrested Taxol-treated NBs did not show microtubule growth or elongation, or mitotic progression (Supplementary Movies 5 and 6). By comparison, microtubules in control NBs in prophase and mitosis showed prominent EB1-EGFP puncta (Fig. 6c) and rapid microtubule growth and elongation (*n* = 18, total = 18) (Supplementary Movies 3 and 4).

Taxol-treated larval brains stained for α-tubulin (*n* = 8 BLs, *n* = 4 tVNCs, *n* = 4 LBs) showed stabilized microtubule bundles in the neuropil that extended into NSC protrusions, necks, and cell bodies (Supplementary Fig. 3). Microtubule spikes from the neuropil into NSC protrusions were also observed in BLs and tVNCs without Taxol treatment, but microtubules were not as bundled as following Taxol treatment (Fig. 3a–d and Supplementary Fig. 3). The Taxol-induced bundled microtubules in NSC protrusions, necks, and cell bodies parallels the mitochondrial clustering in these NSC regions after Taxol treatment (Fig. 5a, c).

These observations demonstrate that the conditions that we used to treat larvae with Taxol suppress microtubule dynamics in larval brains, blocking microtubule growth and elongation, and bundling microtubules, preventing normal spindle assembly in NBs. Taxol treatment also affects qNSC reactivation—qNSCs can undergo reactivation under the conditions of Taxol treatment that we used, but at significantly reduced frequencies, and the NBs that are formed are delayed or blocked in division. The reduced rate of qNSC activation and suppressed mitotic progression of NBs that are formed presumably delay development and causes the small brain phenotype that we observe for Taxol-treated larvae, although other effects of Taxol on mitosis and its regulation could also contribute to the small brain size.

### Midgut stem cells contain mitochondria-rich protrusions

The finding of clustered mitochondria in *Drosophila* larval brain qNSCs led us to ask whether mitochondria were associated with other stem cells that respond to insulin signaling, e.g., *Drosophila* ISCs^7,37^. ISCs have an apical extension that was first observed by TEM^38^, but it has not been extensively described. We labeled ISCs and their immediate daughter enteroblasts (EBs) in normally cultured or starved then refed (denoted “fed”) females by expressing green fluorescent protein (GFP) driven by the ISC/EB-specific transcription factor, Escargot (Esg). Early enterocytes (ECs), into which EBs differentiate, were also labeled by *esg-gal4*-induced GFP. The GFP in the midgut stem and progenitor cells was localized primarily to the nucleus, but it was also present in the cytoplasm, allowing visualization of the cellular protrusions. We refer to cells showing Esg-specific GFP expression as Esg-positive cells.

**Fig. 7:**
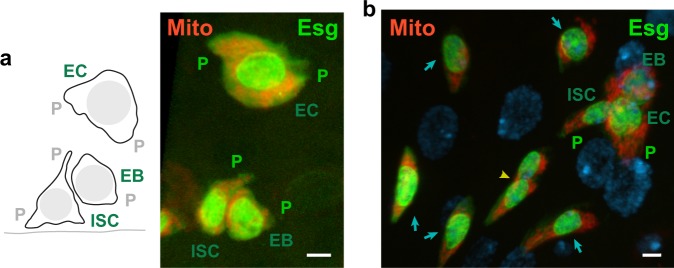
*Drosophila* adult midgut stem and progenitor cells contain protrusions with clustered mitochondria. **a** Intestinal stem and progenitor three-cell clone (left, schematic drawing). The EC is apically positioned relative to the ISC/EB. Line (bottom), basal surface. ISC, intestinal stem cell; EB, enteroblast; EC, early enterocyte; P, protrusion. Esg-positive (Esg, green) midgut three-cell group (right) with inferred ISC, EB, and EC cells expressing mito-RFP (Mito, red) show protrusions (P) packed with mitochondria and mitochondria clustered around the nuclei in cell bodies. Protrusions of putative ISCs ranged from 0.75 to 7.9 µm and averaged 2.7 ± 1.8 µm (mean ± SD, *n* = 22) from the nucleus to the cell membrane and those of putative EBs and ECs were 1.50 ± 0.86 µm (*n* = 12) and 4.04 ± 3.10 µm, (*n* = 22), respectively. The putative ISC protrusions differed significantly in length from the putative EB protrusions (*P* = 0.0337, unpaired *t*-test) but not from the putative EC cytoplasmic extensions (*P* = 0.0838, unpaired *t*-test). **b** Esg-positive, mito-RFP-expressing cells (fed female) were present in midguts as single cells (blue arrows), two-cell groups (yellow arrowhead), or three-cell groups (top right). DAPI-stained mature EC nuclei (diffuse blue spheres). Projections of *z*-sections with 3D surfaces rendered in Imaris (**a**) or without rendering (**b**); view from basal to apical surface (**a**) or from apical to basal surface (**b**). Bars, 3 µm.

Esg-positive cells occurred in the midgut as single cells, or two- or three-cell groups, which were interpreted to correspond to ISCs, ISC/EBs, or ISC/EB/ECs, respectively, based on cell size and apical or basal position relative to one another^38^ (Fig. 7). Cytoplasmic protrusions were present on all three cell types. The putative ISCs showed baso-lateral protrusions and/or apical protrusions that resembled lamellipodia, whereas putative EBs displayed smaller lamellipodia-like protrusions and putative ECs had large cytoplasmic extensions on either side of the cell (Fig. 7).

The Esg-positive cells contained mitochondria clustered around their nuclei in cell bodies and packed, rather than randomly distributed, into apical and basal protrusions. Mitochondrial clusters were observed in all midguts that were imaged. They were found in single cells (*n* = 20) and two-cell (*n* = 20) and three-cell (*n* = 18) groups (total = 58 putative clones, total = 114 cells, *n* = 3 midguts); no cells were observed without mitochondrial clusters (*n* = 0, total = 58 putative clones, total = 114 cells, *n* = 3 midguts). The larger protrusions of inferred ISCs and ECs had correspondingly larger numbers of clustered mitochondria.

The ISC niche has not been conclusively identified in *Drosophila*, but it is thought to comprise the stem cell microenvironment^16,39^, including nearby cells, based on analogies to the mouse, where surrounding cells are believed to contribute to the ISC niche^40^. The ISC protrusions could therefore play roles in sensing environmental cues and communicating with niche components.

## Discussion

The qNSC and ISC cytoplasmic protrusions both form stem-cell-to-niche bridges containing mitochondria along their length, despite their morphological differences—the qNSC protrusions are thin and can extend up to ~45 µm from the cell body to the neuropil, whereas the lamellipodia-like protrusions associated with putative ISCs extend only up to ~8 µm from the nucleus to the cell membrane. In addition, both stem cells contain mitochondria clustered around their nuclei in cell bodies. Mammalian B1 astrocytes and subventricular zone radial glial cells are thought to function as qNSCs or NSCs, and their cytoplasmic extensions, which allow them to contact their niche component, the vasculature^41^, also contain prominent mitochondria as observed by TEM^42,43^. These observations imply that the mitochondria in these stem cells may be needed to preserve the stem-cell state prior to activation. This interpretation is consistent with the finding of a number of mitochondrial genes among those specifically expressed in larval brain qNSCs or adult midgut ISC/EBs^6,44^ (Supplementary Tables 1 and 2).

Mitochondria have been shown previously to affect stem cell quiescence^45^, although the mechanism by which they do so is not known. One proposal is that metabolites produced by mitochondria are intermediates in pathways for chromatin or histone modifications, e.g., acetylation, methylation, or glutarylation^46^, which maintain quiescence or silence gene expression. Larval brain qNSCs and midgut ISCs are sensitive to insulin and undergo activation in its presence. The finding of mitochondria prominently distributed along the thin qNSC protrusions and packed into protrusions of presumed ISCs implies that the protrusions serve as sensors for activation by the insulin pathway. Insulin has been shown to promote microtubule growth^47^, which is likely to disrupt mitochondrial clustering in qNSCs and ISCs, reversing chromatin silencing and initiating stem cell activation or division.

Mitochondria packed around NSC nuclei could mediate quiescence by producing metabolites needed for chromatin silencing and act as sensors for activation by dispersing along protrusions in response to insulin-induced microtubule growth. The enhanced clustering of mitochondria in Taxol-treated qNSCs supports the idea that the mitochondrial clusters in the qNSC neck and cell bodies are maintained by the slow forward-and-backward microtubule movement that we observe by EB1-EGFP tracking, given that suppressing microtubule dynamics by Taxol treatment enhances the clustering and causes microtubule bundles to form that extend into NSC protrusions, necks, and cell bodies. Under the Taxol conditions that we used, the number of activated NBs in larval brains are significantly reduced, consistent with the idea that blocking microtubule dynamics with Taxol suppresses qNSC reactivation. Brains of Taxol-treated larvae are delayed in development and are smaller than normal, consistent with the reduced number of NBs that we observe; Taxol-induced apoptosis^48^ or mitotic delay resulting from spindle checkpoint activation^49^ could also contribute to or cause the brains of Taxol-treated larvae to be smaller.

Thus, larval brain qNSCs and adult midgut ISCs both contain clustered mitochondria in their protrusions and cell bodies, and are activated by insulin. These features are shared by mammalian qNSC or NSCs—they may also be relevant to other stem and progenitor cells that are sensitive to insulin. The protrusions themselves represent an unusual class of cytoplasmic extensions that differ from those previously known in that they form stem-cell-to-niche mitochondrial bridges that may play an important role in both stem cell quiescence and activation.

## Methods

### *Drosophila* stocks and molecular reagents

The following *Drosophila* lines were obtained from colleagues, the Bloomington Stock Center (BDSC) or Kyoto Stock Center (Kyoto DGRC): *insc*-*gal4* (BDSC #8751), *esg*-*gal4 UAS::GFP P{tubP-Gal80*^*ts*^*}* (Bruce Edgar, University of Utah), *UAS-CD8::GFP* (BDSC #32186), *UAS-EB1::EGFP* (BDSC #36861), *UAS-tdTomato::Cox8A* (Kyoto DGRC #117015 and #117016), *Ubq-YFP::Asl*^28^, *P{ncd-γTub37C.GFP}F13F3* (BDSC #56831)^30^, and *Ubq-αTub::GFP*^50^.

The following Abs, listed with Research Resource Identifiers (RRIDs), were used in this study: Guinea pig polyclonal anti-Dpn Ab (J. Skeath, RRID: AB_2314299), Rabbit monoclonal anti-TOMM20 Ab (Abcam, Cat #ab186734; RRID: AB_2716623), Mouse monoclonal anti-Mira mAb (F. Matsuzaki, Riken CDB, Kobe JP), Mouse monoclonal anti-αTub-rhodamine mAb (W. Sullivan, University of California, Santa Cruz, CA USA), Rabbit polyclonal anti-γ-tubulin Ab (Y. Zheng, Carnegie Institution for Science, Baltimore, MD, USA), Rabbit polyclonal anti-RFP pAb (MBL Life Science, Cat #PM005; RRID: AB_591279), Goat anti-guinea pig-Alexa 568 (ThermoFisher/Molecular Probes, Cat #A-11075; RRID: AB_141954), Donkey anti-guinea pig-Alexa 488 (Jackson ImmunoResearch Laboratories, Inc., Cat #706-545-148; RRID: AB_2340472), Donkey anti-guinea pig-Alexa 647 (Jackson ImmunoResearch Laboratories, Inc., Cat #706-605-148: RRID: AB_2340476), Donkey anti-mouse-Alexa 647 (Jackson ImmunoResearch Laboratories, Inc., Cat #715-605-150: RRID: AB_2340862), Goat anti-rabbit-Alexa 647 (Molecular Probes/ThermoFisher, 1:500), and Goat anti-rabbit-Alexa 568 (ThermoFisher/Molecular Probes, Cat #A-11011; RRID: AB_143157).

### TEM specimen preparation and imaging

Embryos were collected for 1–4 h from a *y w* line cultured on standard medium at room temperature (RT). After washing in deionized distilled water, embryos were transferred to amino-acid-depleted medium (5% sucrose, 1× phosphate-buffered saline (PBS), 1% agar) to hatch, followed by 20–28 h to develop prior to dissection. The qNSCs of larvae hatched on amino-acid-depleted medium do not undergo reactivation and NBs are smaller than those in age-matched wild-type larvae raised on standard food. Under the conditions we used, qNSCs are enriched in the larval brains—activated NBs are also present due to carryover of yeast and soluble amino acids with the embryos onto the sucrose–PBS–agar plates.

First instar larval brains for electron microscopy (EM) were dissected under fixative (4% EM-grade glutaraldehyde, 1x PBS, pH ~ 7) at RT and collected into fixative in small microfuge tubes, then stored in fixative at 4 °C. Dissections were performed on stereomicroscopes placed in front of a chemical fume hood with full exhaust turned on and the sash opened to the position of maximum air intake to draw away fixative fumes, together with appropriate personal protective equipment (PPE; high filtration face masks, safety glasses, gloves, and laboratory coats), using small volumes of fixative (25 µl) to dissect three to five larvae before replenishing. A tube of ~135 larval brains was divided into two samples, each was pelleted and embedded in agarose and the two agarose blocks were processed separately. Post-fixation staining with tannic acid and OsO_4_, dehydration, and embedding in resin were essentially as described^51^. Thick (~0.5 µm) and thin (60–70 nm) sections were cut from both blocks for observation. Thin sections were stained with uranyl acetate/lead citrate prior to scanning on a CM12 TEM (ThermoFisher) equipped with an AMT digital camera to record images.

### Fluorescence microscopy

Brains were dissected from larvae hatched and maintained ~6–8 h on standard food at RT or hatched and maintained 20–24 h on amino-acid-depleted medium at RT before dissection. Because of their small size, larval brains were fixed and stained while still attached to mouth hooks and pieces of cuticle; brains were collected into 20 µl of fixative in the cap of an inverted 0.2 ml PCR tube affixed with double-stick tape to a microscope slide. Solutions were removed with a drawn-out glass microliter pipette. Fixation was in 4% EM-grade formaldehyde + 1× PHEM (60 mM Pipes, 25 mM HEPES, 2 mM MgSO_4_, 10 mM EGTA, pH 7.2) for ~20–30 min at RT, followed by washes in 0.3% PBST (PBS + 0.3% Triton-X). Appropriate PPE was used during fixation, as described above, to avoid skin contact with or inhalation of fixative. Larval brains were blocked in 3% bovine serum albumin in 0.3% PBST and incubated with primary Ab in blocking buffer at RT. After washing, brains were incubated with secondary Ab and washed; final dissections were performed in 50% glycerol in PBS followed by mounting in 90% glycerol in PBS with double-stick tape and/or vacuum grease pillars as coverslip spacers. Images were acquired on a Zeiss 780 LSM (Duke University Light Microscopy Core Facility, LMCF) using a ×40/1.3 NA EC Plan-Neofluar Oil or ×40/1.4 NA Plan-Apochromat Oil DIC M27 objective.

Images of fixed larval brains after expression of YFP-Asl and CD8-GFP, which were also stained with Abs against Dpn (labeled with Alexa 647 secondary Abs) and α-tubulin (conjugated to NHS-rhodamine), were acquired using the 488 nm line of the Zeiss 780 LSM Ar laser, together with 561 diode laser and 633 nm He/Ne laser lines. The (E)YPF-Asl fluorescence emission peak is shifted by ~20 nm from that of CD8-(E)GFP; the YPF-Asl fluorescence was brighter and appeared more yellow than CD8-GFP fluorescence under the conditions we used for expression and imaging, allowing the YFP-Asl centrosome fluorescence to be distinguished from the CD8-GFP membrane protein fluorescence without spectral unmixing. Staining with anti-γ-tubulin Abs^29^ and labeling with γTub-GFP^30^ were also performed to confirm YFP-Asl localization to qNSC centrosomes. γTub-(S65T)GFP was present as fluorescent puncta throughout the BL and tVNC when imaged alone in live larval brains; it appeared as small paired or single bright spheres of a lighter shade of green when imaged together with CD8-GFP, as shown in Fig. 3e.

Live imaging of EB1-EGFP in brains from larvae hatched on standard or amino-acid-depleted medium was performed after dissection in PBS or Schneider’s medium +10% fetal calf serum (FCS), without or with 20 µg/ml insulin (*LB medium). There was no discernable effect of the dissecting and mounting medium on EB1-EGFP puncta motility—good motility was observed for larval brains from starved larvae dissected and mounted in PBS, and activated asymmetrically dividing NBs were observed in the larval brains, presumably due to the carryover of nutrients with the embryos onto the sucrose–PBS–agar plates, which was greater for *UAS-CD8::GFP insc-gal4* embryos than those not containing *UAS-CD8::GFP*. Larval brains were mounted under a coverslip on an oxygen-permeable Teflon membrane covering the opening of a metal slide^52^. Live imaging was performed on a Bio-Rad Radiance2100 confocal microscope equipped with a Kr/Ar laser and a ×40/1.3 NA EC Plan-Neofluar Oil objective, or a Zeiss 780 LSM equipped with an Ar laser and a ×40/1.4 NA Plan-Apochromat Oil DIC M27 objective.

Midguts were dissected in PBS from adult females raised on standard food and shifted to 29 °C for 4–7 days to induce mito-RFP expression, and immediately fixed in 3.7% paraformaldehyde + 0.3% PBST for 30 min. In some experiments, females were starved for 24 or 48 h, then fed on standard food supplemented with yeast paste for 24 h before midguts were dissected and fixed; these females are referred to as “fed.” Immunostaining was performed to enhance mito-RFP, then midguts were mounted in Vectashield (Vector Laboratories, Inc.) for imaging. Images were recorded on a Zeiss AxioImager M.2 using ApoTome structured illumination optical sectioning and a ×63/1.4 NA Plan-Apochromat Oil objective, or a Zeiss 510 LSM (Duke University LMCF) equipped with a ×63/1.4 NA Plan-Apochromat Oil DIC objective.

### Taxol treatment

*UAS-CD8::GFP insc-gal4/UAS-tdTomato::Cox8A* embryos were collected overnight on normal food and ~80–100 embryos were transferred to a small improvised culture dish (2.7 cm ID) of grape juice agar (5.6% glucose, 2.8% sugar, 1.7% yeast, 2.1% agar, 43.5% grape juice; pH ~ 4.2, adjusted with 42% proprionic acid + 3.6% phosphoric acid after autoclaving). Excess moisture was removed by blotting with filter paper strips, then 80 µl of 100 µM Taxol in PBS were added to the embryos on the surface of the plate. After 18–20 h at RT, an additional 40 µl of 100 µM Taxol in PBS were added to the embryos and larvae on the plate. Following a further 21–22 h, larvae, 2 days after hatching and delayed in late L1/L2, were collected and transferred to a small piece of grape juice agar in an inverted microfuge cap and incubated in 10 µl of 100 µM Taxol in PBS for 2–3 h. Larvae were then transferred to LB medium (Schneider’s *Drosophila* medium, 10% FCS, 20 µg/ml insulin, 40 µg/ml glutathione, 2 mM glutamine) + 100 µM Taxol for 20–30 min before dissecting and mounting in LB medium + Taxol for imaging. For viability test controls, embryos (~80–100/test) were transferred to grape juice agar plates without Taxol to hatch and develop.

Larvae for imaging controls were hatched on normal food, then L1 larvae during the first day after hatching were dissected and mounted in LB medium or Schneider’s *Drosophila* medium + 10% FCS for imaging. Quantification of images acquired with the two media showed no significant differences in maximum fluorescence (LB medium, 158.5 ± 12.2 au, mean ± SEM, *n* = 8; Schneider’s medium +10% FCS, 171.8 ± 7.9 au, *n* = 12; unpaired *t*-test, *P* = 0.35).

*UAS-CD8::GFP insc-gal4/UAS-EB1::EGFP* embryos to test for microtubule dynamics were treated with Taxol as described above, but the final incubation was in *LB medium (LB medium without glutathione or glutamine) + 100 µM Taxol for 20 min before dissecting and mounting in *LB medium + Taxol for imaging. Control larvae were hatched on normal food, then dissected and mounted in *LB medium for imaging.

### Imaging and image analysis

Immunofluorescence (IFM) and fluorescence images were adjusted for brightness and contrast in ImageJ/FIJI^53^ or Adobe PhotoShop. Linear adjustments were applied to the entire image corresponding to a single channel.

Kymographs were made from time-lapse sequences acquired by live imaging and displacements were measured in Adobe Illustrator and/or ImageJ/FIJI. Velocities were calculated from the displacements and time stamps recorded with the images.

Imaging of mitochondria (mito-RFP) and CD8-GFP in fixed fed vs. starved or live Taxol-treated vs. control BLs for quantification of fluorescence was performed under identical settings of laser power, gain, scan speed, averaging, and pinhole settings on the same day or the next day, using the same objective, zoom, and laser-scanning confocal microscope to acquire *z*-section stacks of 3 µm depth images. Quantification of mito-RFP fluorescence was performed in ImageJ. BLs in two to four sections ≥3 µm from the cortex were measured to obtain the minimum and maximum fluorescence, total fluorescence, and the area. Images that were quantified did not contain saturated pixels.

The relative size of Taxol-treated and control BLs was estimated by modeling the BLs as spheres of volume, *V* = ^4^/_3_ π *r*^*3*^ and the *z*-sections as circles of area, *A* = π *r*^*2*^. The radius of Taxol-treated (*r*_T_) and control (*r*_C_) BLs was calculated from the area measured from the *z*-section in which the BL was the largest. This gave *A*_T_ = 3208.3 ± 572.4 µm^2^ (mean ± SD, *n* = 5 BLs) and *A*_C_ = 4855.8 ± 1389.8 µm^2^ (*n* = 7 BLs) for Taxol-treated (*A*_T_) and control (*A*_C_) BLs, respectively, corresponding to *r*_T_ = 32.2 ± 2.7 µm and *r*_C_ = 39.0 ± 5.5 µm, and *r*_T_/*r*_C_ = 0.826. The relative volume of Taxol-treated (*V*_T_) and control (*V*_C_) BLs was then calculated by *V*_T_*/V*_C_ = *r*_T_^3^*/r*_C_^3^, giving 0.563.

ApoTome or confocal *z*-section image stacks of adult female midguts were projected in Imaris v 9.2.0 and surfaces were rendered to create ISC/EB/EC three-dimensional models. Nuclear and cytoplasmic Esg-induced GFP fluorescence was built into separate surfaces to visualize cytoplasmic protrusions more easily. Surfaces corresponding to Esg-induced GFP and mito-RFP fluorescence were made partially transparent to make underlying cellular features visible. Imaris *z*-section projections without surface rendering were made to analyze relative position of ISC/EB/EC cells to ascertain their inferred identity.

Mitochondrial clusters were analyzed in ApoTome *z*-section midgut image stacks, using FIJI Max Intensity Z projections as a guide. Clustering was defined as >3 adjacent mito-RFP foci, together with >5 mito-RFP foci per cell.

### Bioinformatics

*Drosophila melanogaster* mitochondrial genes were compiled by searching Flybase Release FB2019_01 Feb 22, 2019 using gene ontogeny (GO) terms “mitochondrion” and “ER-mitochondrion membrane contact site”. The compiled mitochondrial genes were used to search genes specifically expressed in *Drosophila* larval brain qNSCs^6^ or ISC/EBs^44^. After eliminating duplicates, Metascape was used to perform GO Biological Processes enrichment analysis.

### qNSC and midgut stem cell identification

Larval brain qNSCs were identified in TEM images by their small size (~2–4 µm diameter) and characteristic morphology, as described in the text. Briefly, qNSCs contain dark heterochromatic nuclear patches with a prominent nucleolus under the staining conditions that we used; they also have irregular cell margins, scant cytoplasm, and a single protrusion. By contrast, glial cells show pale nuclear staining^24^, contain more cytoplasm, and have several processes, giving them a stellate appearance. qNSCs were identified in fixed larval brains by immunostaining with Dpn, a neural progenitor-specific, Hairy and Enhancer-of-Split transcription factor^54^, and the presence of a cellular protrusion, delineated by neural progenitor-specific *insc-gal4-*induced^35^ expression of CD8-GFP, a membrane marker^55^, or Mira, a neural progenitor scaffold protein involved in NB asymmetric division^56^. For larval brain live imaging, *insc-gal4*-induced CD8-GFP was used to identify NSCs with a cellular protrusion.

Adult midgut stem and progenitor cells were identified by Esg-induced expression of GFP. ISCs, EBs, and early ECs were tentatively identified by their occurrence as Esg-positive single cells or in two- or three-cell groups, and their relative size and basal/apical position to one another, as described previously^38^. Adult female midguts were used for imaging, but ISCs are also observed in males^38^ and are expected to show the same cytological features.

### Statistics and reproducibility

GraphPad Prism was used to evaluate data for statistical differences using *χ*^2^-tests or unpaired *t*-tests and to make box-and-whisker plots of the data. The plots show boxes that extend from the 25th to 75th percentiles of the data, a line within the box at the median, and bars or whiskers that mark the minimum and maximum values.

TEM was performed on a sample of ~135 larval brains from starved conditions to enrich for qNSCs. Because of the labor involved in sample preparation and the TEM preparation time and costs, the mitochondrial clustering in qNSCs that we observed by TEM was confirmed by IMF and fluorescence analysis. The IMF analysis did not show noticeable differences in mitochondrial clustering in fed and starved LBs and analysis of mito-RFP fluorescence intensity in fed and starved LBs (Supplementary Fig. 2) showed no significant differences. These findings indicate that fed and starved LBs are similar in their mitochondrial levels and distribution.

Fixed brains from fed or starved larvae to confirm mitochondrial clustering in qNSCs were prepared in two or more separate experiments for each genotype that was analyzed; staining experiments for fed (five tests) or starved (six tests) conditions were performed on samples of three to seven fixed LBs using mito-RFP or anti-TOMM20 Ab to label mitochondria. IFM staining experiments to demonstrate that qNSCs contain centrosomes were performed using YFP-Asl (2 tests, *n* = 6 LBs) or anti-γTub Ab (4 tests, *n* = 6 LBs) to label centrosomes; the presence of centrosomes in qNSCs was confirmed by live imaging of γTub-GFP LBs and Ab staining of fixed γTub-GFP LBs. Live imaging of EB1-GFP in NSCs to analyze microtubule growth was performed on 7 days (total = 23 LBs). Live imaging of mito-RFP motility in NSCs was also performed (11 days, total = 28 LBs), but is not reported here because of variable results. Taxol experiments, which each required 3 days to complete, were performed two to three times for each genotype that was analyzed to determine the drug effects on mitochondrial distribution (mito-RFP, *n* = 11 LBs; control, *n* = 6 LBs), microtubule growth (EB1-GFP, *n* = 9 LBs; control, *n* = 14 LBs), and microtubule bundling (anti-αTub-rhodamine mAb, *n* = 7 LBs; control, *n* = 3 LBs). Microtubule bundling was also analyzed in fixed Taxol-treated LBs expressing αTub-GFP (*n* = 5 LBs; control, *n* = 5 LBs). Fluorescence measurements were made on different optical slices of BLs, where the number of individual BLs was *n* = 3–7.

Where possible, immunostaining or fluorescence localization experiments were performed with different Abs or GFP-labeled proteins directed against the same cytological target, e.g., anti-Mira mAb and CD8-GFP were used to label qNSC protrusions; anti-TOMM20 Ab and mito-RFP were used to detect mitochondria; YFP-Asl, γTub Ab, γTub-GFP, and EB1-GFP were used to identify qNSC centrosomes; and anti-αTub-rhodamine mAb, αTub-GFP, and EB1-GFP were used to visualize bundled microtubules in Taxol-treated larval brains.

Midguts were prepared in three separate experiments from mixed age females (*n* = 10), starved (24 h, *n* = 10) and fed females (24 h starved/24 h fed, *n* = 11), and starved (48 h, *n* = 5) and fed females (48 h starved/24 hr fed, *n* = 3). Esg-positive cells with mitochondria-enriched protrusions were observed in all midguts that were imaged.

### Reporting summary

Further information on research design is available in the Nature Research Reporting Summary linked to this article.

## Supplementary information

Supplementary Information

Description of Additional Supplementary Files

Supplementary Data 1

Supplementary Movie 1

Supplementary Movie 2

Supplementary Movie 3

Supplementary Movie 4

Supplementary Movie 5

Supplementary Movie 6

Reporting Summary

## Data Availability

All datasets generated and analyzed during the current study are available from the corresponding author on reasonable request. Data are currently being stored on high-capacity computers and external hard drives.
