# Supplementary material for: Mitochondria-enriched protrusions are associated with brain and intestinal stem cells in Drosophila
Source: Commun Biol. 2019 Nov 22;2:427. doi: 10.1038/s42003-019-0671-4 (PMC6874589; doi:10.1038/s42003-019-0671-4)
Supplement: Supplementary file 1 — Supplementary Information [file 42003_2019_671_MOESM1_ESM.pdf]

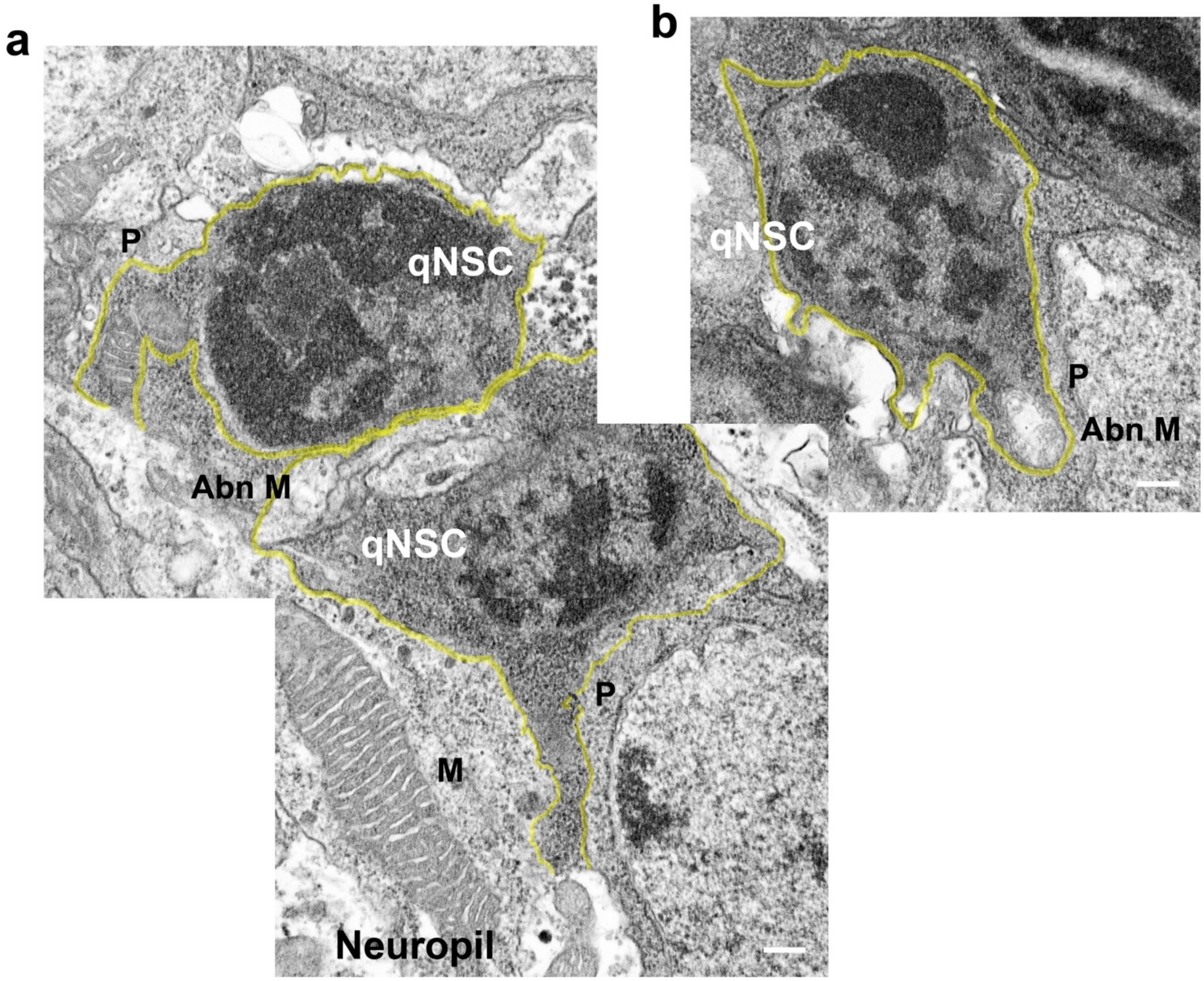

Supplementary Figure 1 qNSCs contain abnormal mitochondria. **a** Mitochondria with distorted cristae (Abn M, abnormal mitochondria) in a qNSC cell body (see Fig. 1g). **b** Abnormal mitochondrium in a qNSC protrusion (P) with a vacuole and abnormal cristae. Bars, 200 nm.

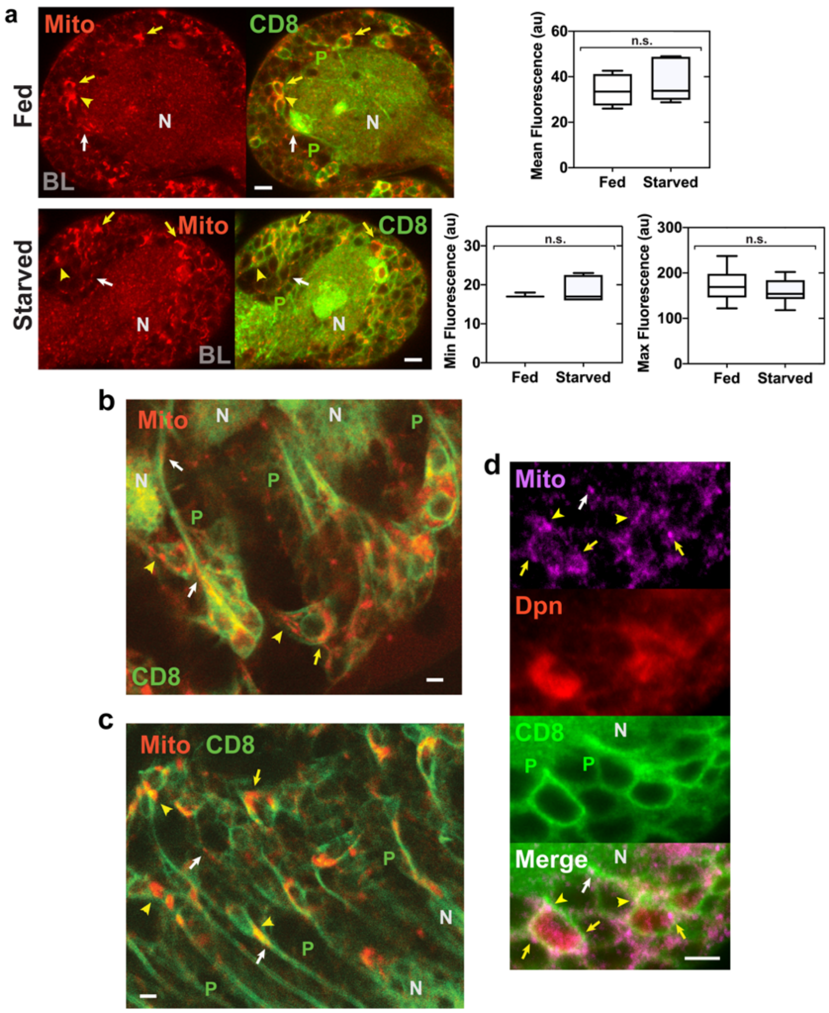

Supplementary Figure 2 qNSC protrusions in both fed and starved larvae contain clustered mitochondria. **a** Mitochondria (Mito, mito-RFP, red) in fed (top) and starved (bottom) fixed larval BL z-sections showed no significant difference in mito-RFP mean intensity (representative images; plot, Mean Fluorescence, right; au, arbitrary units; n.s., not significant) or distribution (plots, Mininum, Min Fluorescence; Maximum, Max Fluorescence, right). Mean fluorescence: fed, 34.0±2.1 au, *mean±SEM*, *n*=9 z-sections, *n*=3 BLs; starved, 37.5±2.9 au, *n*=9 z-sections, *n*=3 BLs; *P*=0.35, unpaired *t* test. Minimum fluorescence: fed, 17.1±0.1 au, *n*=9 z-sections, *n*=3 BLs; starved, 18.6±1.0 au, *n*=9 z-sections, *n*=3 BLs; *P*=0.19, unpaired *t* test. Maximum fluorescence: fed, 173.1±11.6 au, *n*=9 z-sections, *n*=3 BLs; starved,160.2±8.8 au, *n*=9 z-sections, *n*= 3 BLs; *P*=0.39, unpaired *t* test. Mitochondria in NSC protrusions (white arrows), necks (yellow arrowheads) and cell bodies (yellow arrows). CD8, CD8 membrane protein (green); BL, brain lobe; N, neuropil; P, protrusion. Bars, 6 μm. **b** Mito-RFP (red) in live BL NSCs and **c** live tVNC NSCs (fed larvae) (see Fig. 2c,e for higher magnification images). **d** Mitochondria in BL qNSCs immunostained for TOMM20 mitochondrial protein (Mito, purple; starved larva). IFM images showing all channels (see Fig. 2f right, for other overlays). Dpn (red), Deadpan. Bars (**b,c,d**), 3 μm.

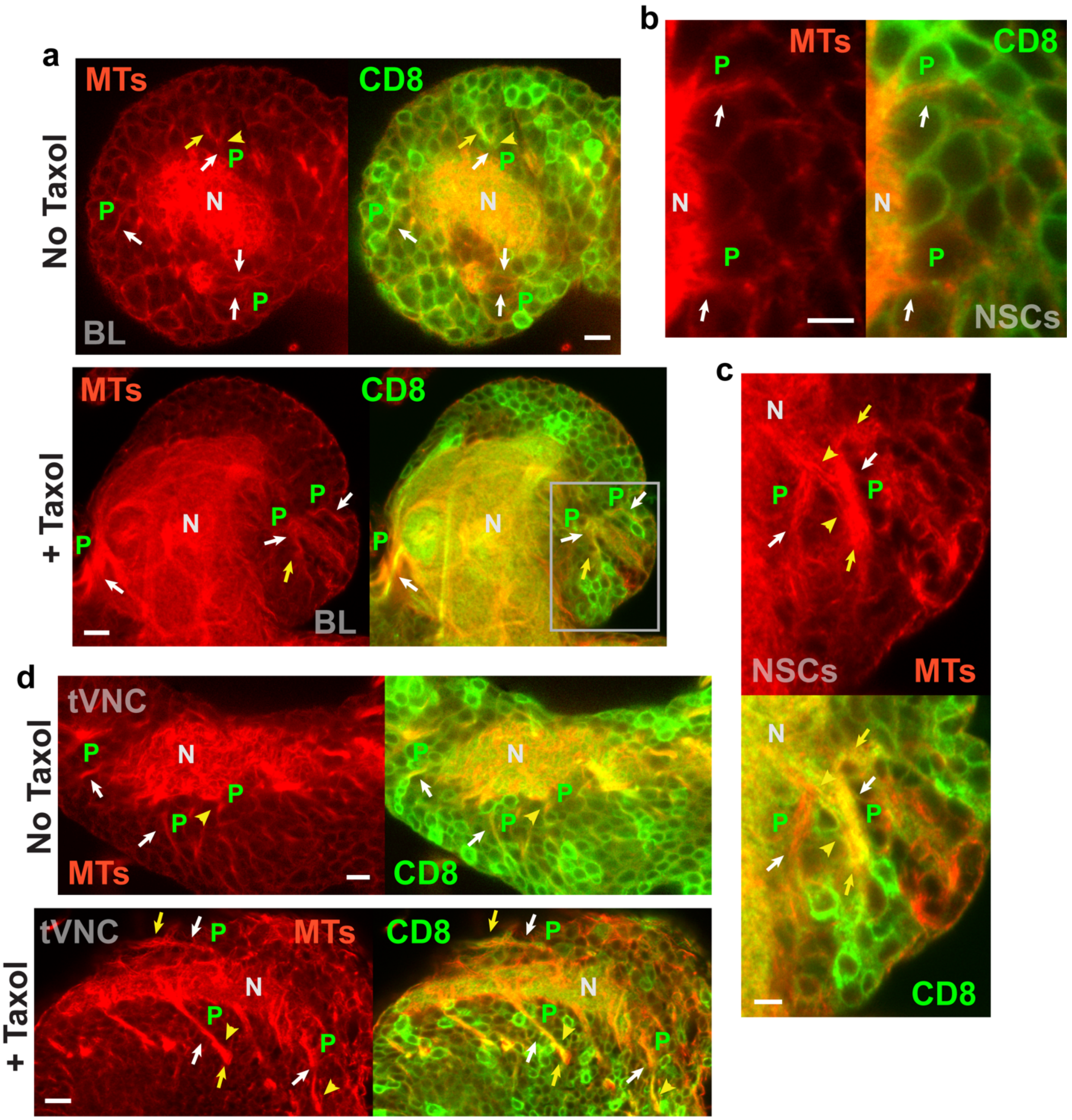

Supplementary Figure 3 Taxol stabilizes and bundles microtubules in larval brains. **a** Microtubules (MTs, rhodamine-labeled αTub mAb, red) in a control (No Taxol, top) and Taxol-treated (+Tx, bottom) brain lobe (BL), and **b** control and **c** Taxol-treated neural stem cells (NSCs; **c** higher magnification image from **a**, inset, gray box, plane of focus in **c** differs slightly from boxed region) showing enhanced bundling after Taxol treatment. NSC protrusions (P, white arrows), necks (yellow arrowheads) and cell bodies (yellow arrows). N, neuropil. CD8, CD8 membrane protein (green). **d** Microtubules in a control (top) or Taxol-treated thoracic ventral nerve cord (tVNC; bottom); bundled microtubules in the neuropil extend into tVNC NSC protrusions, necks and cell bodies after Taxol treatment. Bars: 6 μm (**a**, **d**); 3 μm (**b**,**c**).

Supplementary Table 1 qNSC-specific mitochondrial genes.

No	Gene name	No	Gene name		Gene name	No	Gene name	No	Gene name
1	Hsc70-4	21	Kaz1-ORFB	41	ND-ASHI	61	kdn	81	LysRS
2	ND-MWFE	22	GstZ1	42	Gpo3	62	CG3902	82	Men-b
3	CG11077	23	ND-B14.5B	43	CG12118	63	whd	83	Hsp68
4	CG3719	24	ppl	44	Hsc70-5	64	mino	84	Trxr-1
5	mRpL55	25	Coq4	45	Menl-2	65	Got1		
6	Tim8	26	CG15093	46	Menl-1	66	ND-ACP		
7	CG17991	27	Fis1	47	Gpo1	67	Oat		
8	arg	28	ND-19	48	Dhfr	68	Pdk		
9	CG15390	29	Etf-QO	49	Adck	69	Got2		
10	Nap1	30	Mccc2	50	Idh3a	70	spoon		
11	ND-B15	31	ND-B17.2	51	CG4860	71	Shmt		
12	Tom7	32	Nmdmc	52	Idh3b	72	mRpS30		
13	CG32281	33	ND-13B	53	pr	73	cav		
14	mRpL53	34	CG14806	54	pnut	74	Aldh		
15	CG11752	35	CG6638	55	Nipsnap	75	CysRS-m		
16	Gdh	36	Pdha	56	Baldspot	76	Hsc70-1		
17	Adk2	37	Dcp-1	57	CG1640	77	CG5214		
18	Chchd3	38	Men	58	muc	78	zuc		
19	Sirup	39	RFeSP	59	slgA	79	Gdap1		
20	Rpp20	40	Ssadh	60	larp	80	CG7461		

Annotated list of mitochondrial genes among genes expressed in *Drosophila* late embryo/early larval qNSCs identified in screen by Otsuki and Brand[1].

Gene enrichment by Metascape

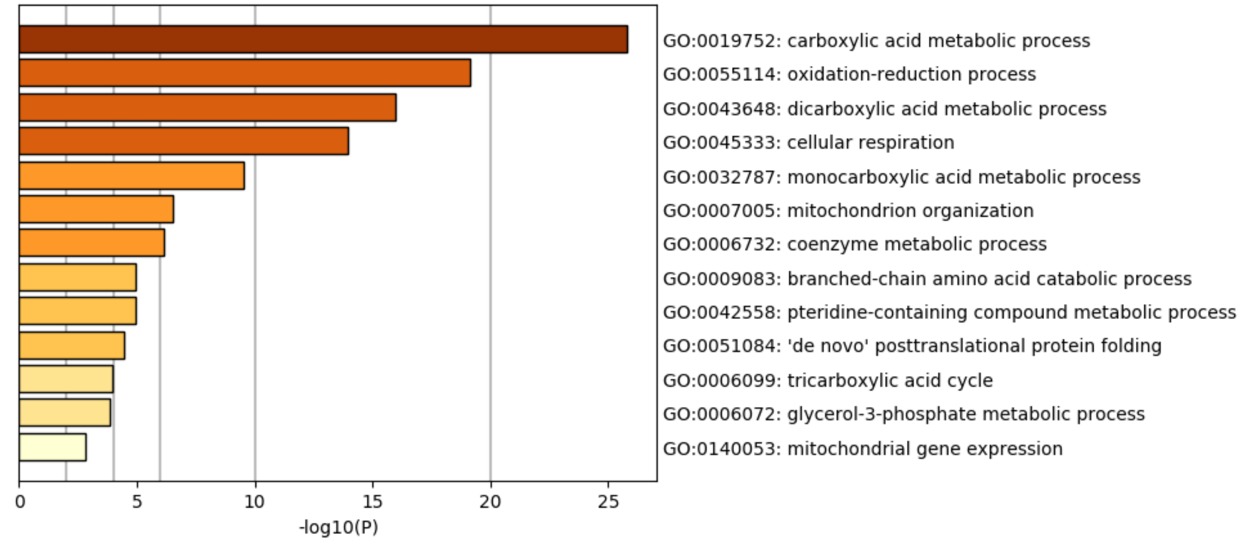

Gene ontology (GO) terms for qNSC-specific mitochondrial genes.

GO	Category	Description	Count	%	Log10(P)	Log10(q)
GO:0019752	GO Biological Processes	carboxylic acid metabolic process	33	39.29	-25.78	-21.89
GO:0055114	GO Biological Processes	oxidation-reduction process	31	36.90	-19.16	-15.87
GO:0043648	GO Biological Processes	dicarboxylic acid metabolic process	12	14.29	-16.00	-12.80
GO:0045333	GO Biological Processes	cellular respiration	15	17.86	-13.98	-10.99
GO:0032787	GO Biological Processes	monocarboxylic acid metabolic process	13	15.48	-9.51	-6.82
GO:0007005	GO Biological Processes	mitochondrion organization	11	13.10	-6.52	-4.09
GO:0006732	GO Biological Processes	coenzyme metabolic process	9	10.71	-6.13	-3.75
GO:0009083	GO Biological Processes	branched-chain amino acid catabolic process	3	3.57	-4.95	-2.74
GO:0042558	GO Biological Processes	pteridine-containing compound metabolic process	4	4.76	-4.92	-2.72
GO:0051084	GO Biological Processes	'de novo' posttranslational protein folding	4	4.76	-4.47	-2.36
GO:0006099	GO Biological Processes	tricarboxylic acid cycle	4	4.76	-3.96	-1.92
GO:0006072	GO Biological Processes	glycerol-3-phosphate metabolic process	3	3.57	-3.86	-1.84
GO:0140053	GO Biological Processes	mitochondrial gene expression	5	5.95	-2.82	-0.88

Supplementary Table 2 ISC/EB-specific mitochondrial genes.

No	Gene name	No	Gene name	No	Gene name	No	Gene name
1	Dhap-at	21	CG5599	41	mRF1	61	mRpL22
2	CG8993	22	CG11110	42	ND-49	62	RNaseZ
3	CG43998	23	Mthfs	43	CG5037	63	l(2)37Cc
4	CG43999	24	nmdyn-D7	44	CG14407	64	Pdp
5	CG14043	25	mRpS26	45	Slc25A46a	65	mEFG1
6	mRpL32	26	mRpL46	46	CG13295	66	mRpL50
7	mRpL45	27	GstZ1	47	CG3021	67	mTerf5
8	Tim17b	28	CG44194	48	mRRF1	68	Las
9	hid	29	AsnRS-m	49	HisRS	69	CG17141
10	dnk	30	ND-24	50	CG3107	70	Exd2
11	iPLA2-VIA	31	Ppox	51	mRpS28	71	MICU1
12	Fdx1	32	mRpL51	52	dare	72	Miga
13	cav	33	mRpS23	53	mRpL49	73	Mer
14	Rae1	34	Sccpdh1	54	TyrRS-m	74	Spag1
15	ND-B18	35	GlyRS	55	ScsbetaG	75	ttm2
16	CG42376	36	DNApol-gamma35	56	Coq8	76	GLS
17	ND-B8	37	CG1307	57	CG10932	77	clu
18	CG3156	38	mRpS17	58	Pgam5	78	arg
19	Egm	39	ND-13A	59	CG34242	79	shd
20	CG3776	40	CG11815	60	CG18528		

Annotated list of mitochondrial genes among genes with enriched expression in *Drosophila* ISC/EBs identified in screen by Doupé et al.[2].

Gene enrichment by Metascape.

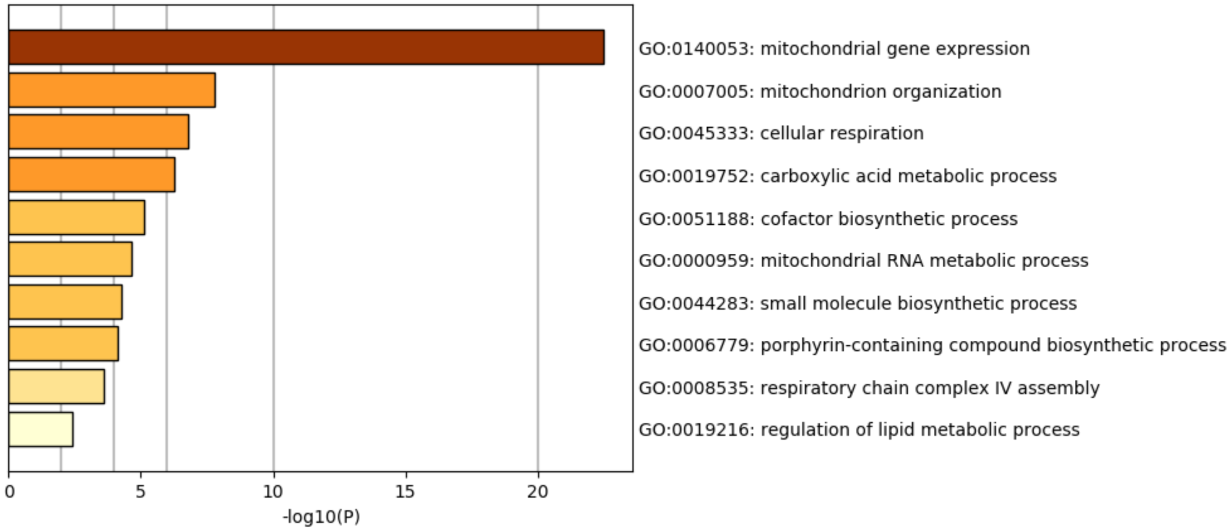

Gene ontology (GO) terms for ISC/EB-specific mitochondrial genes.

GO	Category	Description	Count	%	Log10(P)	Log10(q)
GO:0140053	GO Biological Processes	mitochondrial gene expression	**20**	25.32	-22.47	-18.58
GO:0007005	GO Biological Processes	mitochondrion organization	**12**	15.19	-7.80	-4.39
GO:0045333	GO Biological Processes	cellular respiration	**9**	11.39	-6.79	-3.60
GO:0019752	GO Biological Processes	carboxylic acid metabolic process	**14**	17.72	-6.24	-3.13
GO:0051188	GO Biological Processes	cofactor biosynthetic process	**7**	8.86	-5.14	-2.36
GO:0000959	GO Biological Processes	mitochondrial RNA metabolic process	**4**	5.06	-4.64	-1.98
GO:0044283	GO Biological Processes	small molecule biosynthetic process	**9**	11.39	-4.28	-1.82
GO:0006779	GO Biological Processes	porphyrin-containing compound biosynthetic process	**3**	3.80	-4.13	-1.72
GO:0008535	GO Biological Processes	respiratory chain complex IV assembly	**3**	3.80	-3.62	-1.41
GO:0019216	GO Biological Processes	regulation of lipid metabolic process	**3**	3.80	-2.42	-0.38

Supplementary References

1. Otsuki, L. & Brand, A. H. Cell cycle heterogeneity directs the timing of neural stem cell activation from quiescence. *Science* **360**, 99-102 (2018).

2. Doupé, D. P., Marshall, O. J., Dayton, H., Brand, A. H. & Perrimon, N. *Drosophila* intestinal stem and progenitor cells are major sources and regulators of homeostatic niche signals. *Proc. Natl Acad. Sci. USA* **115**, 12218-12223 (2018).
