# Supplementary material for: Mitochondria-enriched protrusions are associated with brain and intestinal stem cells in Drosophila
Source: Commun Biol. 2019 Nov 22;2:427. doi: 10.1038/s42003-019-0671-4 (PMC6874589; doi:10.1038/s42003-019-0671-4)
Supplement: Supplementary file 2 — Description of Additional Supplementary Files [file 42003_2019_671_MOESM2_ESM.docx]

**Description of Additional Supplementary Items**

Additional supplementary items include the following:

**Supplemental Data 1 (source data)**

File containing data for the scatter plot in Fig. 2 and box-and-whisker plots in Fig. 4-6 and Supplementary Figure 2

**Supplementary Movie 1**

Microtubule growth in NSCs

**Supplementary Movie 2**

Microtubule growth in an activated asymmetrically dividing NB

**Supplementary Movie 3**

Microtubule dynamics in an activated non-Taxol treated NB during prophase

**Supplementary Movie 4**

Microtubule dynamics in an activated non-Taxol treated NB during mitosis

**Supplementary Movie 5**

Microtubule dynamics in an activated Taxol-treated NB during prophase

**Supplementary Movie 6**

Microtubule dynamics in an activated Taxol-treated NB during mitosis
