# Supplementary material for: Mitochondria-enriched protrusions are associated with brain and intestinal stem cells in Drosophila
Source: Commun Biol. 2019 Nov 22;2:427. doi: 10.1038/s42003-019-0671-4 (PMC6874589; doi:10.1038/s42003-019-0671-4)
Supplement: Supplementary file 10 — Reporting Summary [file 42003_2019_671_MOESM10_ESM.pdf]

nature research

Corresponding author(s): Sharyn A Endow

Last updated by author(s): 11/01/2019 v2

Reporting Summary

Nature Research wishes to improve the reproducibility of the work that we publish. This form provides structure for consistency and transparency in reporting. For further information on Nature Research policies, see Authors & Referees and the Editorial Policy Checklist.

Statistics

For all statistical analyses, confirm that the following items are present in the figure legend, table legend, main text, or Methods section.

n/a	Confirmed	
☐	☒	The exact sample size (n) for each experimental group/condition, given as a discrete number and unit of measurement
☐	☒	A statement on whether measurements were taken from distinct samples or whether the same sample was measured repeatedly
☐	☒	The statistical test(s) used AND whether they are one- or two-sided<br>*Only common tests should be described solely by name; describe more complex techniques in the Methods section.*
☒	☐	A description of all covariates tested
☒	☐	A description of any assumptions or corrections, such as tests of normality and adjustment for multiple comparisons
☒	☐	A full description of the statistical parameters including central tendency (e.g. means) or other basic estimates (e.g. regression coefficient) AND variation (e.g. standard deviation) or associated estimates of uncertainty (e.g. confidence intervals)
☒	☐	For null hypothesis testing, the test statistic (e.g. F, t, r) with confidence intervals, effect sizes, degrees of freedom and P value noted<br>*Give P values as exact values whenever suitable.*
☒	☐	For Bayesian analysis, information on the choice of priors and Markov chain Monte Carlo settings
☒	☐	For hierarchical and complex designs, identification of the appropriate level for tests and full reporting of outcomes
☒	☐	Estimates of effect sizes (e.g. Cohen's d, Pearson's r), indicating how they were calculated

Our web collection on statistics for biologists contains articles on many of the points above.

Software and code

Policy information about availability of computer code

Data collection	NA
Data analysis	NA

For manuscripts utilizing custom algorithms or software that are central to the research but not yet described in published literature, software must be made available to editors/reviewers. We strongly encourage code deposition in a community repository (e.g. GitHub). See the Nature Research guidelines for submitting code & software for further information.

Data

Policy information about availability of data

All manuscripts must include a data availability statement. This statement should provide the following information, where applicable:

- Accession codes, unique identifiers, or web links for publicly available datasets
- A list of figures that have associated raw data
- A description of any restrictions on data availability

All datasets generated and analyzed during the current study are available from the corresponding author on reasonable request. Data are currently being stored on high-capacity computers and external hard drives. Correspondence and requests for materials should be addressed to endow@duke.edu

Field-specific reporting

Please select the one below that is the best fit for your research. If you are not sure, read the appropriate sections before making your selection.

☒ Life sciences ☐ Behavioural & social sciences ☐ Ecological, evolutionary & environmental sciences

Life sciences study design

All studies must disclose on these points even when the disclosure is negative.

Sample size

No sample-size calculations were performed. Experiments were performed until samples repeatedly gave the same result and/or experiments using different biological reagents (e.g., FP-labeled proteins vs antibodies) gave the same results. These sample sizes were considered to be sufficient from the large number of samples showing the same result, e.g, n=19-25 qNSCs in IFM experiments to detect mitochondria in fed or starved BLs and n=27-35 qNSCs in IFM experiments to detect centrosomes in BLs. Statistical analysis was used in some experiments as a measure of a sufficiently large sample size, e.g., to calculate a P value to determine whether the number of activated neuroblasts was significantly reduced after Taxol treatment.

Data exclusions

Data were excluded from analysis if there was unacceptably high background for immunofluorescence (IMF) experiments or clear mislocalization of antibody staining, or consistent variability in repeated experiments. In the case of IMF, our initial tests of some antibodies showed high nonspecific background such that we were not able to reliably analyze the results - the IMF experiments were repeated with FP-labeled proteins in combination with antibodies and gave consistent results that are included in the ms. Antibody mislocalization was observed with an alpha-tubulin Ab under conditions in which the antibody solution was reused from a previous staining - this resulted in clear mislocalization to the nucleus rather than the cytoplasm (microtubules are not known to be present in the nucleus but are present in the cytoplasm) and was attributed to a trace component in the previous sample that altered antibody specificity. The data were reproduced by using only freshly diluted (not reused) antibody solutions and also by analysis of a FP-labeled alpha-tubulin protein (instead of antibody staining) in the experiment. Live imaging of mito-RFP (mitochondrial) motility was attempted numerous times (11 days, n=28 larval brains) but the data were not included in the ms because of the variable results.

Replication

TEM was performed on a sample of ~135 larval brains from starved conditions to enrich for qNSCs, but was not repeated because of the labor involved in sample preparation and the TEM preparation time and costs. Instead, the mitochondrial clustering in qNSCs that we observed by TEM was confirmed by immunofluorescence (IMF) and fluorescence analysis. The IMF analysis did not show noticeable differences in mitochondrial clustering in fed and starved LBs and analysis of mito-RFP fluorescence intensity in fed and starved LBs (Supplementary Figure 2) showed no significant differences in mean, minimum or maximum fluorescence. These findings indicate that fed and starved LBs are similar in their mitochondrial levels and distribution. Fixed brains from fed or starved larvae to confirm mitochondrial clustering in qNSCs were prepared in two or more separate experiments for each genotype that was analyzed; staining experiments for fed (5 tests) or starved (6 tests) conditions were performed on samples of 3-7 fixed LBs using mito-RFP or anti-TOMM20 Ab to label mitochondria. Immunofluorescence staining experiments to demonstrate that qNSCs contain centrosomes were performed using YFP-Asl (2 tests, n=6 LBs) or anti-gammaTub Ab (4 tests, n=6 LBs) to label centrosomes; the presence of centrosomes in qNSCs was confirmed by live imaging of gammaTub-GFP LBs and antibody staining of fixed gammaTub-GFP LBs. Live imaging of EB1-GFP in NSCs to analyze microtubule growth was performed on 7 days (total=23 LBs). Taxol experiments, which each required 3 days to complete, were performed 2-3 times for each genotype that was analyzed to determine the drug effects on mitochondrial distribution (mito-RFP, n=11 LBs; control, n=6 LBs), microtubule growth (EB1-GFP, n=9 LBs; control, n=14 LBs) and microtubule bundling (anti-alphaTub-rhodamine mAb, n=7 LBs; control, n=3 LBs). Microtubule bundling was also analyzed in fixed Taxol-treated LBs expressing alphaTub-GFP (n=5 LBs; control, n=5 LBs). The numbers refer to different tests or LBs. Fluorescence measurements were made on different optical slices of BLs, where the number of individual BLs was n=3-7.

Where possible, immunostaining or fluorescence localization experiments were performed with different antibodies or GFP-labeled proteins directed against the same cytological target, e.g., anti-Mira mAb and CD8-GFP were used to label qNSC protrusions; anti-TOMM20 Ab and mito-RFP were used to detect mitochondria; YFP-Asl, gammaTub-GFP and EB1-GFP were used to identify qNSC centrosomes; and anti-alphaTub-rhodamine mAb, alphaTub-GFP and EB1-GFP were used to visualize bundled microtubules in Taxol-treated larval brains.

Midguts were prepared in three separate experiments from mixed age females (n=10); starved (24 hr, n=10) and fed females (24 hr starved/24 hr fed, n=11); and starved (48 hr, n=5) and fed females (48 hr starved/24 hr fed, n=3). Esg-positive cells with mitochondria-enriched protrusions were observed in all midguts that were imaged.

Randomization

Covariates were not relevant to our experimental studies - the studies that were performed were analyzed for the effects being studied and showed clear results, e.g., the Taxol treatments clearly blocked microtubule dynamics by anti-alphaTub staining, and alphaTub-GFP and EB1-GFP analysis, and showed greatly increased mitochondrial clustering in the NSCs.

Blinding

None of the experiments that were conducted were subjected to blinded analysis - the studies that were performed gave clear results, e.g., the Taxol treatments clearly blocked microtubule dynamics by anti-alphaTub staining, and alphaTub-GFP and EB1-GFP analysis, and showed greatly increased mitochondrial clustering in the NSCs.

Reporting for specific materials, systems and methods

We require information from authors about some types of materials, experimental systems and methods used in many studies. Here, indicate whether each material, system or method listed is relevant to your study. If you are not sure if a list item applies to your research, read the appropriate section before selecting a response.

Materials & experimental systems

n/a	Involved in the study	
☐	☒	Antibodies
☒	☐	Eukaryotic cell lines
☒	☐	Palaeontology
☐	☒	Animals and other organisms
☒	☐	Human research participants
☒	☐	Clinical data

Methods

n/a	Involved in the study	
☒	☐	ChIP-seq
☒	☐	Flow cytometry
☒	☐	MRI-based neuroimaging

Antibodies

Antibodies used	The following antibodies (Ab), listed with Research Resource Identifiers (RRIDs), were used in this study: Guinea pig polyclonal anti-Dpn Ab (J. Skeath, RRID: AB_2314299), Rabbit monoclonal anti-TOMM20 Ab (Abcam, Cat #ab186734; RRID: AB_2716623), Mouse monoclonal anti-Mira mAb (F. Matsuzaki, JP), Mouse monoclonal anti-alpha-Tub-rhodamine mAb (W. Sullivan, USA), Rabbit polyclonal anti-gamma-tubulin Ab (Y. Zheng, USA), Rabbit polyclonal anti-RFP pAb (MBL Life Science, Cat #PM005; RRID: AB_591279), Goat anti-guinea pig-Alexa 568 (ThermoFisher/Molecular Probes, Cat #A-11075; RRID: AB_141954), Donkey anti-guinea pig-Alexa 488 (Jackson ImmunoResearch Laboratories, Inc., Cat #706-545-148; RRID: AB_2340472), Donkey anti-guinea pig-Alexa 647 (Jackson ImmunoResearch Laboratories, Inc., Cat #706-605-148: RRID: AB_2340476), Donkey anti-mouse-Alexa 647 (Jackson ImmunoResearch Laboratories, Inc., Cat #715-605-150: RRID: AB_2340862), Goat anti-rabbit-Alexa 647 (Molecular Probes/ThermoFisher, 1:500), Goat anti-rabbit-Alexa 568 (ThermoFisher/Molecular Probes, Cat #A-11011; RRID: AB_143157).
Validation	RRIDs are given as validation for primary Abs listed above, except for anti-Mira Ab, which has been validated by Oshiro et al. 2000 Nature 408, 593-596 and other publications, anti-alpha-Tub-rhodamine mAb, which has been validated by Endow et al. 1994 J Cell Sci 107, 859-867 and other publications, and anti-gamma-tubulin Ab, which has been validated by Zheng et al. 1995 Nature 378, 587-583 and other publications.

Animals and other organisms

Policy information about studies involving animals; ARRIVE guidelines recommended for reporting animal research

Laboratory animals	The following Drosophila lines were obtained from colleagues, the Bloomington Stock Center (BDSC) or Kyoto Stock Center (Kyoto DGRC): insc-gal4 (BDSC #8751), esg-gal4 UAS::GFP P{tubP-Gal80ts} (B. Edgar, USA), UAS-CD8::GFP (BDSC #32186), UAS-EB1::GFP (BDSC #36861), UAS-tdTomato::Cox8A (Kyoto DGRC #117015 and #117016), Ubq-YFP::Asl (C. Gonzalez, Spain), P{ncd-gammaTub37C.GFP}F13F3 (BDSC #56831), Ubq-alphaTub::GFP (C. Gonzalez, Spain). Adult female midguts were used for imaging, but intestinal stem cells are also observed in males and are expected to show the same cytological features.
Wild animals	NA
Field-collected samples	NA
Ethics oversight	NA

Note that full information on the approval of the study protocol must also be provided in the manuscript.
